# Elimination of receptor binding by influenza hemagglutinin improves vaccine-induced immunity

**DOI:** 10.1038/s41541-022-00463-3

**Published:** 2022-04-11

**Authors:** Hilary E. Hendin, Pierre-Olivier Lavoie, Jason M. Gravett, Stéphane Pillet, Pooja Saxena, Nathalie Landry, Marc-André D’Aoust, Brian J. Ward

**Affiliations:** 1 Department of Microbiology and Immunology, McGill University, 3775 University Street, Montréal, QC H3A 2B4 Canada; 2 Research Institute of McGill University Health Centre, Glen Site, 1001 Décarie Boul., Montréal, QC H4AG 3J1 Canada; 3 Medicago Inc., 1020 Route de l’Église, Bureau 600, Québec, QC Canada

**Keywords:** Protein vaccines, Influenza virus

## Abstract

The binding of influenza hemagglutinin (HA) to sialic acid (SA) receptors plays a well-defined role in shaping infection but the impact of such binding on vaccine responses has not yet been explored. We generated a virus-like particle (VLP) vaccine bearing the HA of H1N1 A/California/07/09 that is unable to bind to its α(2,6)-linked SA receptor (H1_*Y98F*_-VLP) and compared its immunogenicity and efficacy to a wild-type H1-VLP (H1_*WT*_-VLP) in mice. The H1_*Y98F*_-VLP elicited significantly stronger and more durable antibody responses (hemagglutination inhibition and microneutralization titers) and greater avidity maturation, likely attributable to improved germinal center formation. H1_*Y98F*_-VLP also resulted in a robust population of IL-2^+^TNFα^+^IFNγ^−^ CD4^+^ T cells that correlated with antibody responses. Compared to H1_*WT*_-VLP vaccination, mice immunized with H1_*Y98F*_-VLP had 2.3-log lower lung viral loads and significantly lower pulmonary inflammatory cytokine levels 5 days post-challenge. These findings suggest that abrogation of HA-SA interactions may be a promising strategy to improve the quality and durability of influenza vaccine-induced humoral responses.

## Introduction

Vaccination is widely recognized as the most effective way to prevent influenza infection and to reduce the societal and economic burden of seasonal influenza epidemics. However, influenza vaccines have remained largely unchanged since their introduction in the mid-1940s^1,2^ despite significant year-to-year and strain-to-strain variation in effectiveness^3,4^. This inconsistency raises doubts about whether current vaccines would be suitably effective in the context of a pandemic and highlights the need for novel and more reliable approaches to influenza vaccination.

Influenza hemagglutinin (HA) is a trimeric glycoprotein on the surface of all influenza viruses that initiates infection by binding to sialic acid (SA) receptors on the surface of respiratory epithelial cells^5^. Antibodies that bind to the receptor-binding domain (RBD) of HA can block this initial interaction with the host cell and are the basis for the most widely used correlate of protection for influenza infection^6,7^. HA proteins are therefore the major and, in some cases, the only antigens in all commercial influenza vaccines^8^. The binding properties of influenza HA are strain-specific and have been extensively studied in the context of disease severity and transmissibility^5,9–11^. However, the implications of HA binding properties on influenza vaccine immunogenicity and efficacy have not been investigated despite the presence of SA receptors on the surface of cells throughout the body^12–14^.

We previously demonstrated that the strain-specific binding properties of influenza HA influence the pattern of interaction of HA-bearing virus-like particles (VLP) with human peripheral blood mononuclear cells (PBMC) in vitro^15^. These interactions were driven by differential expression of α2,3- and α2,6-linked SA on human PBMC and strongly influenced downstream innate immune activation^15^, raising the possibility that the binding properties of HA may be important modulators of influenza vaccine immunogenicity.

In the current work, we sought to determine whether HA-SA interactions influence vaccine responses in vivo. We generated a plant-based HA-VLP vaccine bearing H1 (A/California/07/2009) that was unable to bind SA and compared its immunogenicity and efficacy to the wild-type (WT) H1-VLP using a murine model. Mutation of H1 binding was achieved by a single amino acid substitution from tyrosine to phenylalanine at residue 98 (Y98F, H3 numbering), which prevents HA-SA interactions by eliminating the hydroxyl group required for hydrogen bonding with SA. This mutation was originally described by Martín et al. using H3 (A/Aichi/2/68)^16^ but has subsequently been shown to prevent SA binding of many influenza A HAs, including H1 (A/California/07/2009), due to its position within a conserved region of the RBD^17^. This mutation does not affect the integrity of the RBD or HA folding^17,18^ making the H1_*Y98F*_-VLP a good candidate for studying the possible impact of HA-SA interactions on vaccine responses. Herein, we demonstrate that elimination of HA-SA interactions significantly improves both the immunogenicity and efficacy of a plant-based H1-VLP vaccine.

## Results

### Generation and validation of H1_*Y98F*_-VLP

**Fig. 1:**
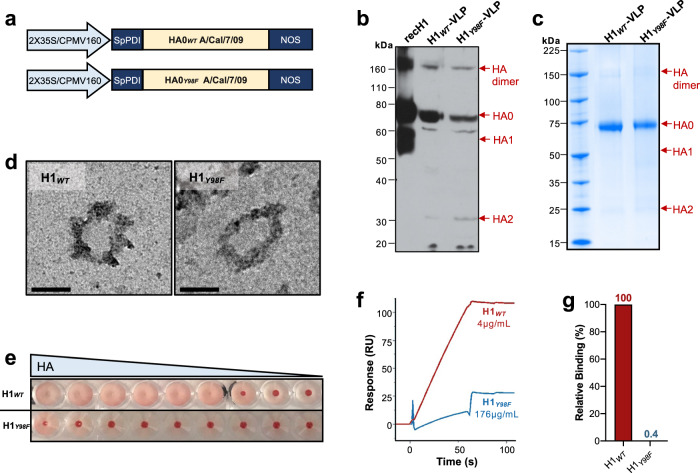
Y98F mutation abrogates SA binding without affecting HA expression or VLP structure. Wild-type (WT) H1 (H1_*WT*_) and Y98F H1 (H1_*Y98F*_) were expressed in *Nicotiana benthamiana*. **a** Representation of the H1_*WT*_ (top) and H1_*Y98F*_ (bottom) expression cassettes. 2X35S/CPMV160 promoter, double 35S promoter fused to the 5′ UTR of a cowpea mosaic virus (CPMV) expression enhancer; SpPDI signal peptide from alfalfa protein disulfide isomerase, NOS nopaline synthase terminator signal. **b** Expression of HA was confirmed by SDS-PAGE of crude leaf extracts followed by immunoblot analysis. Commercially available recombinant H1 expressed in HEK-293 cells (recH1, Immune Technologies) was included as a positive control (1 µg). 1° ab: rabbit polyclonal anti-H1 1:500 (Cat. No. IT-003-SW, Immune Technology); 2° ab: horseradish peroxidase-conjugated goat anti-rabbit IgG 1:20000 (Cat. No. IT-200-01, Immune Technology). **c** VLP composition and purity were evaluated by SDS-PAGE of purified leaf digests followed by Coomassie G-250 staining. **d** Representative TEM images show the similar size and morphology of H1_*WT*_- and H1_*Y98F*_-VLP. Images were acquired using a Tecnai G2 Spirit transmission electron microscope. Scale bar = 100 nm. **e** Sialic acid (SA) binding was evaluated based on hemagglutination of turkey red blood cells following incubation (30 min) with serial two-fold dilutions of H1_*WT*_- and H1_*Y98F*_-VLP (starting at 1:150 and 1:10, respectively). SA binding was further quantified by SPR. **f** SPR sensorgram showing the binding response of H1_*WT*_- (red) and H1_*Y98F*_-VLP (blue) to α-2,6 SA captured on a streptavidin-coated chip surface. **g** Relative binding of H1_*Y98F*_-VLP when adjusted for HA content.

VLP composed of a plant-lipid bilayer studded with the WT H1 (H1_*WT*_-VLP) or Y98F H1 (H1_*Y98F*_-VLP) were expressed in *Nicotiana benthamiana* as previously described using a 2X35S/CPMV160/NOS expression system^19–21^ (Fig. 1a). Expression of HA in crude leaf digests was confirmed by western blot (Fig. 1b). Following purification, VLP preparations were separated by gel electrophoresis and visualized using Coomassie G-250 staining to evaluate the protein composition and purity (Fig. 1c). HA was predominantly expressed in its uncleaved form (HA0), however, faint bands corresponding to cleaved HA (HA1 and HA2) and HA dimers were observed. The purity of the HA-VLP products was determined by analysis of the densitometry profile of each protein band and was comparable between formulations (~95%). VLP size (~100 nm) and morphology were consistent with previous reports^20,22^ and were unaffected by the Y98F mutation (Fig. 1d).

With multiple active HA trimers on their surface, H1_*WT*_-VLPs are capable of mediating hemagglutination by binding to SA on the surface of red blood cells (RBC). Introduction of the Y98F mutation prevented hemagglutination, indicating that SA binding was greatly reduced or abolished (Fig. 1e). Furthermore, H1_*Y98F*_-VLP failed to agglutinate human PBMC (Supplementary Fig. 1a). Incubation of PBMC with H1_*Y98F*_-VLP dramatically reduced polyclonal B cell activation that occurs following binding of the H1_*WT*_-VLP^15,23^ and increased T cell activation (Supplementary Fig. 1b–d), indicating that reduced binding can modulate downstream immune responses. To confirm that the Y98F mutation inhibits HA-SA interactions on a molecular level, we measured binding of H1_*WT*_- and H1_*Y98F*_-VLP to immobilized SA by surface plasmon resonance (SPR). The slope of the association curve was 1.66 resonance units per second (RU/s) for H1_WT_-VLP and 0.3 RU/s for H1_*Y98F*_-VLP (Fig. 1f). Importantly, H1_*Y98F*_-VLP was tested at a much higher concentration than H1_*WT*_-VLP (44x). When the slopes were adjusted for HA content, a 99.6% reduction in binding was attributable to the Y98F mutation (H1 0.59 RU/s; Y98F H1 0.002 RU/s) (Fig. 1g). Taken together, these data suggested that H1_*Y98F*_-VLP was an appropriate tool to evaluate the role of HA-SA interactions in the context of influenza vaccine responses.

### H1_*Y98F*_ -VLP elicits stronger and more durable humoral responses

To establish whether HA-SA interactions influence the humoral immune response to vaccination in mice, we measured the development of H1-specific antibodies in sera following vaccination with 1 dose of H1_*WT*_-VLP or H1_*Y98F*_-VLP (3 µg HA/dose) or an equivalent volume of PBS (placebo). Total H1-specifc IgG was measured by ELISA and functional antibodies were quantified using the hemagglutination inhibition (HI) assay to measure antibodies that block the binding of live virus to avian RBCs^24^ and the microneutralization (MN) assay to measure antibodies that prevent infection of Madin–Darby canine kidney (MDCK) cells^25^. Antibodies were quantified at 21 days post-vaccination (dpv) to characterize pre-challenge humoral responses and on a monthly basis (1–3 m, 7 m) to evaluate the kinetics and durability of the antibody responses.

**Fig. 2:**
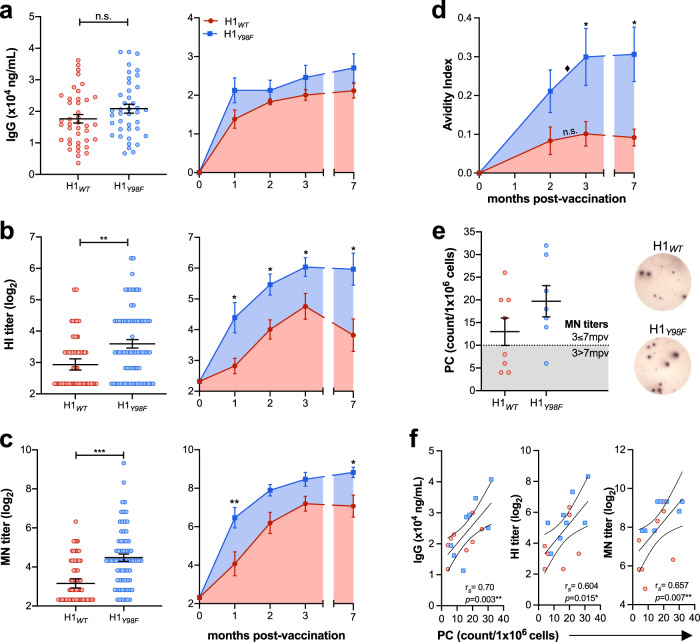
H1_*Y98F*_-VLP elicits a more robust and durable antibody response. Mice were vaccinated (IM) with H1_*WT*_- or H1_*Y98F*_-VLP (3 µg/dose). Sera were collected at day 21 (left panel) or on a monthly basis (right panel) to measure (**a**) total H1-specific IgG by ELISA, (**b**) hemagglutination inhibition titers and (**c**) microneutralization titers. **d** Avidity indices of sera obtained 2–7 mpv following incubation with 8 M urea. **e** H1-specific IgG-producing plasma cells (PC) in the bone marrow measured by ELISpot. Representative wells from each group are shown on the right. **f** Spearman’s rank correlation technique was applied to evaluate the relationship between the frequency of PC and IgG titers (left), HI titers (middle) and MN titers (right). Error bars represent the mean ± SEM. At day 21, *N* = 40–70/group and data are pooled from six independent experiments. For long-term studies, *N* = 7–8/group. Statistical significance between vaccine groups was determined by Mann–Whitney test (*p < 0.033, **p < 0.01, ***p < 0.001). Statistical significance between time points within the same group was determined by two-way ANOVA with the Geisser-Greenhouse correction and Sidak’s multiple comparisons (^♦^*p* < 0.033).

Immunization with H1_*WT*_-VLP or H1_*Y98F*_-VLP resulted in comparable H1-specific IgG titers by ELISA at all time points (Fig. 2a) but there were marked differences in antibody functionality. Most notably, vaccination with H1_*Y98F*_-VLP resulted in significantly higher HI and MN titers at 21 dpv (*p* = 0.002 and *p* < 0.001, respectively) (Fig. 2b–c). Titers increased until 3 months post-vaccination (mpv) in both groups but were consistently higher among mice vaccinated with H1_*Y98F*_-VLP. Furthermore, H1_*Y98F*_-VLP resulted in improved durability of HI titers, which declined dramatically between 3 and 7 mpv in mice vaccinated with H1_*WT*_-VLP. As a result, HI titers were >4-fold higher among mice vaccinated with H1_*Y98F*_-VLP at 7 mpv (*p* = 0.029) (Fig. 2b). MN titers were better maintained in the H1_*WT*_-VLP group at 7 mpv but still declined in 4/8 animals and remained significantly lower than in mice vaccinated with H1_*Y98F*_-VLP (*p* = 0.029) (Fig. 2c). H1-specific antibodies were not detected in the placebo group.

Vaccination with H1_*Y98F*_-VLP also resulted in better IgG avidity maturation. Although H1-specific IgG titers plateaued around 2 mpv, IgG avidity significantly increased between 2 and 3 mpv in mice vaccinated with H1_*Y98F*_-VLP (*p* = 0.03). As a result, IgG avidity was ~3-fold higher in the H1_*Y98F*_-VLP group at 3 mpv and this difference was maintained until 7 mpv (*p* = 0.014) (Fig. 2d). No avidity maturation occurred beyond 2 mpv in mice vaccinated with H1_*WT*_-VLP.

Sustained production of high avidity IgG is thought to be mediated by plasma cells (PC) residing in the bone marrow (BM)^26^. To evaluate whether these cells contribute to the improved durability of antibody responses in mice vaccinated with H1_*Y98F*_-VLP, we quantified H1-specific IgG-producing BM PC at 7 mpv by enzyme-linked immune absorbent spot (ELISpot) assay. Few H1-specific BM PC were detected in the placebo group (2 ± 1 PC/1 × 10^6^ cells). H1-specific BM PC were detected in both vaccine groups and the frequency was higher among mice vaccinated with H1_*Y98F*_-VLP, although this difference did not reach statistical significance (Fig. 2e). Interestingly, the frequency of H1-specific BM PC seemed to correspond with the durability of antibody responses: MN titers declined between 3 and 7 mpv in all mice with <10 PC/1 × 10^6^ BM cells and were maintained in animals with ≥10 PC/1 × 10^6^ BM cells regardless of vaccine group (Fig. 2e). Furthermore, the frequency of H1-specific BM PC significantly correlated with IgG (*r*_*s*_ = 0.704, *p* = 0.003), HI (*r*_*s*_ = 0.604, *p* = 0.015) and MN titers (*r*_*s*_ = 0.657, *p* = 0.007) at 7 mpv (Fig. 2f).

Although adults typically receive a single dose of inactivated influenza vaccine each year, children who are immunologically naïve to influenza require two doses for an adequate immune response^27^. Because laboratory mice are immunologically naive, we evaluated the humoral responses in mice given two doses of H1_*WT*_-VLP or H1_*Y98F*_-VLP administered 21 days apart. By 28 days post-boost, HI titers were significantly higher among mice vaccinated with H1_*Y98F*_-VLP (*p* = 0.02) (Supplementary Fig. 2a) and a similar trend was observed in MN titers (Supplementary Fig. 2b). While no significant differences were observed regarding H1-specific IgG titers (Supplementary Fig. 2c) immunization with two doses of H1_*Y98F*_-VLP resulted in significantly higher IgG avidity (Supplementary Fig. 2d, *p* = 0.03 at 4 M and 6 M urea) and H1-specific IgG-producing PC in the BM by 28 days post-boost (*p* = 0.02) (Supplementary Fig. 2e). Once again, the frequency of BM PC was strongly correlated with IgG titers, HI titers, MN titers, and IgG avidity in both vaccine groups (Supplementary Fig. 2f). Improved humoral responses were also observed among mice vaccinated with two doses of a plant-based non-binding H1_*Y98F*_-VLP based on the H1 A/Idaho/07/2018 sequence (Supplementary Fig. 3a–c) and human cell culture-produced H1_*Y98F*_ trimers based on the H1 A/Brisbane/02/2018 sequence (Supplementary Fig. 4), suggesting that HA-SA interactions broadly impact humoral responses to H1.

### H1_*Y98F*_-VLP improves germinal center reactions

**Fig. 3:**
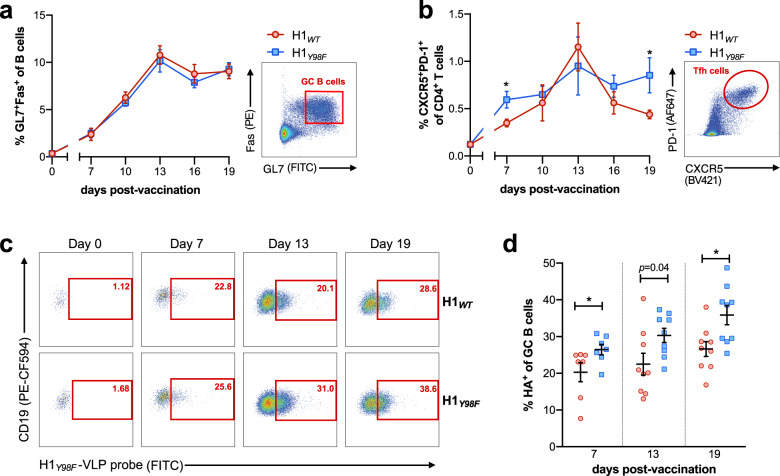
H1_*Y98F*_-VLP promotes enhanced germinal center selection. Mice were immunized with 0.5 µg H1_*WT*_- or H1_*Y98F*_-VLP in the right hind limb footpad and popliteal lymph nodes (pLN) were harvested at indicated time points. The mean frequency (±SEM) of (**a**) CD19^+^Fas^+^GL7^+^ GC B cells and (**b**) CD3^+^CD4^+^CXCR5^+^PD-1^+^ T_FH_ cells was determined by flow cytometry. Representative plots showing GC B cell and T_FH_ gating are shown on the right. To identify H1-specific GC B cells, freshly isolated cells were incubated with 1 µg/ml H1_*Y98F*_-VLP (30 min, 4 °C) and cognate GC B cells were detected following staining with anti-H1 FITC. **c** Representative plots and **d** mean frequency (±SEM) of HA^+^ cells among GC B cells. Data are pooled from three independent experiments, *n* = 7–13/group at each time point. Statistical significance between groups at each time point was determined by Mann–Whitney test (*p < 0.033).

The germinal center (GC) is central to the development of high avidity antibodies and long-lived PC. To determine whether differences in the GC account for improved antibody responses to H1_*Y98F*_-VLP, we evaluated GC kinetics in the draining popliteal lymph node (LN) following footpad injection of H1_*WT*_- or H1_*Y98F*_-VLP. The frequencies of GC B cells (CD19^+^GL7^+^Fas^+^) and T_FH_ cells (CD3^+^CD4^+^CXCR5^+^PD-1^+^) were evaluated by flow cytometry at 3 day intervals 7–19 dpv (see Supplementary Fig. 5 for full gating strategy). Both VLPs resulted in similar frequencies of GC B cells at all time points and peaked at 13 dpv (Fig. 3a). However, striking differences in T_FH_ frequencies and kinetics were observed (Fig. 3b). H1_*Y98F*_-VLP resulted in more rapid induction of T_FH_ cells that were maintained until 19 dpv. In contrast, the H1_*WT*_-VLP resulted in a gradual expansion of T_FH_ cells until 13 dpv followed by a rapid decline. As a result, the frequency of T_FH_ cells was significantly higher among H1_*Y98F*_-VLP-vaccinated mice in early and late GCs (7 dpv *p* = 0.017; 19 dpv *p* = 0.03). No increase in GC B cells or T_FH_ cells was observed at any time point among control mice injected with PBS (Supplementary Fig. 6).

Given the importance of T_FH_ cells in avidity maturation of GC B cells, we next sought to determine whether vaccination with H1_*Y98F*_-VLP results in improved recognition of the H1 antigen among GC B cells. Antigen-specific GC B cells were distinguished based on their ability to bind H1_*Y98F*_-VLP in vitro and were detected using fluorescently labeled anti-H1. This strategy allows for reliable detection of cognate B cells while avoiding SA-mediated interactions with non-cognate cells. H1-specific GC B cells were quantified prior to vaccination and at intervals corresponding to early (7 dpv), peak (13 dpv) and late (19 dpv) GC evolution (Fig. 3c, d). Prior to vaccination GC B cells were rare in all groups (<1% of B cells) and did not bind the H1_*Y98F*_-VLP probe (Fig. 3c). Following vaccination, H1-specific GC B cells were readily detected in both vaccine groups, however, H1_*Y98F*_-VLP resulted in an increased frequency of these cells within the GC at all time points. This effect was most pronounced at 19 dpv when the frequency of H1-specific B cells in the GC was ~30% higher among mice vaccinated with H1_*Y98F*_-VLP compared to H1_*WT*_-VLP (*p* = 0.011) (Fig. 3d). Among non-GC B cells (CD19^+^GL7^−^Fas^−^), the frequency of H1-specific cells was similar between vaccine groups and was comparable to pre-vaccination levels (Supplementary Fig. 7). Taken together, H1_*Y98F*_-VLP favors a more antigen-specific GC response characterized by improved maintenance of T_FH_ cells and expansion of H1-specific GC B cells.

### CD4^+^ T cell response to H1_*Y98F*_-VLP correlates with antibody responses

Antigen-specific CD4^+^ T cells in the spleen and BM were quantified by flow cytometry following vaccination with H1_*WT*_-VLP or H1_*Y98F*_ -VLP. Freshly isolated cells were stimulated with H1_*WT*_-VLP (18 h) or a pool of 131 overlapping peptides (15aa) spanning the H1 sequence (6 h) and responding cells were characterized as antigen-experienced (CD44^+^) CD4^+^ T cells expressing IL-2, IFNγ and/or TNFα (see Supplementary Fig. 8 for full gating strategy). The frequencies of H1-specific CD4^+^ T cells observed following stimulation with either the H1_*WT*_-VLP or the H1 peptide pool were highly correlated (*r*_s_ = 0.5344, *p* < 0.0001) and had similar cytokine signatures (Supplementary Fig. 9). However, the magnitude of the response was greater upon stimulation with the H1_*WT*_-VLP, allowing for more reliable identification of rare populations. Thus, functional signatures were evaluated in cells stimulated with H1_*WT*_-VLP.

**Fig. 4:**
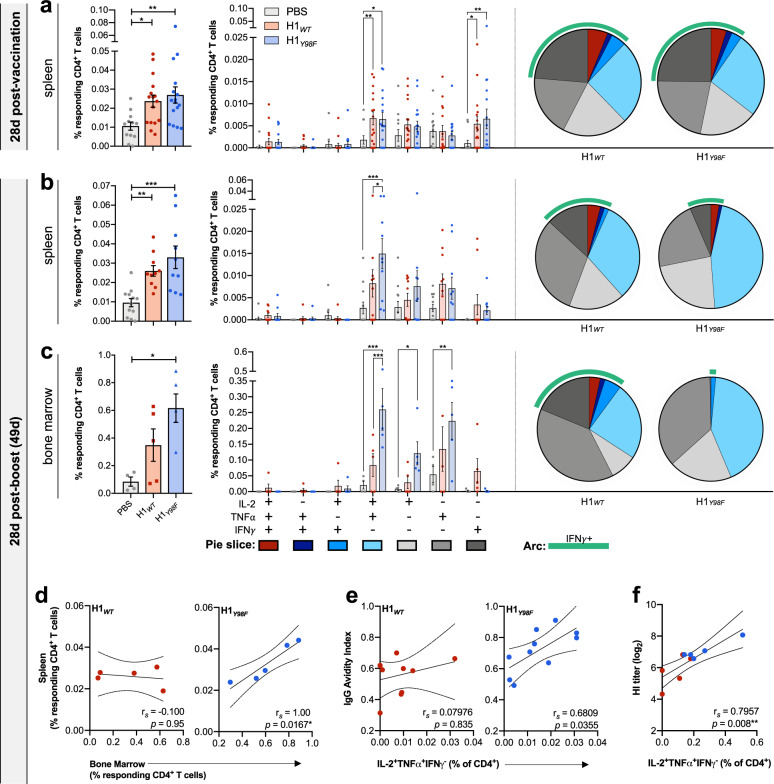
H1_*Y98F*_-VLP elicits robust CD4^+^ T cell responses with enhanced recruitment of antigen-specific CD4^+^ T cells to the bone marrow. Splenocytes and BM immune cells were stimulated for 18 h with 2.5 µg/ml H1_*WT*_-VLP. Flow cytometry was used to quantify H1-specific CD4^+^ T cells in **a** splenocytes isolated at 28 days post-vaccination (3 µg) and in **b** splenocytes and **c** BM immune cells at 28 days post-boost (0.5 µg/dose). Background values obtained from non-stimulated samples were subtracted from values obtained following stimulation with H1_*WT*_-VLP. **a**–**c** The left panel shows the mean frequency (±SEM) of CD4^+^ T cells expressing CD44 and at least one of IL-2, TNFα or IFNγ. The right panel shows the individual cytokine signatures of responding CD4^+^ T cells obtained by Boolean analysis. Color-matched pie charts depict relative distributions of cytokine-producing CD4^+^ T cell populations and IFNγ^+^ populations are highlighted by a green arc. Statistical significance was determined by Kruskal–Wallis test with Dunn’s multiple comparisons (total response) or two-way ANOVA with Tukey’s multiple comparisons (cytokine signatures) (*p < 0.033, **p < 0.01, ***p < 0.001). Spearman’s rank correlation technique was applied to evaluate the relationship between (**d**) the frequency of H1-specific CD4^+^ T cells in the spleen and BM, **e** the frequency of IL-2^+^TNFα^+^IFNγ^-^ CD4^+^ T cells in the spleen and IgG avidity index (4 M urea) and **f** the frequency of IL-2^+^TNFα^+^IFNγ^−^ CD4^+^ T cells in the BM and HI titer (*p < 0.033, **p < 0.01).

A single 3 µg dose of H1_*WT*_-VLP or H1_*Y98F*_-VLP elicited significant populations of H1-specific CD4^+^ T cells in the spleen compared to placebo at 28 days post-vaccination (*p* = 0.01 and *p* = 0.009, respectively). There were no differences in the magnitude or functional signatures between vaccine groups (Fig. 4a). Both vaccines elicited significant populations of IL-2^+^TNFα^+^IFNγ^−^ CD4^+^ T cells (H1_*WT*_ *p* = 0.009; H1_*Y98F*_ *p* = 0.01) and IFNγ single-positive cells compared to the placebo group (H1_*WT*_ *p* = 0.02; H1_*Y98F*_ *p* = 0.002). In contrast, vaccination with two 0.5 µg doses 21 days apart resulted in marked differences in CD4^+^ T cell signatures between vaccine groups (Fig. 4b). In the spleen, the IL-2^+^TNFα^+^IFNγ^−^ population continued to account for the majority of polyfunctional cells. However, this population was significantly larger in mice vaccinated with H1_*Y98F*_-VLP (*p* = 0.01). Conversely, IFNγ^+^ cells were more prevalent among H1-specific CD4^+^ T cells elicited by H1_*WT*_-VLP. It should be noted that the frequency of IFNγ^+^ cells was low in both groups despite substantial IFNγ^+^ populations elicited by a single 3 µg dose of either vaccine. This discrepancy likely reflects the lower antigen dose in the two-dose regimen, as the magnitude of the CD4^+^ T cell response to a plant-based H1_*WT*_-VLP is dose dependent in humans^28^. As expected, IFNγ^+^ and polyfunctional populations were markedly increased in mice that received two 3 µg doses, but functional signatures remained unchanged (Supplementary Fig. 10). With the exception of the frequency and relative proportion of single-positive IL-2 CD4^+^ T cells, similar signatures were also observed following vaccination with H1_*WT*_- and H1_*Y98F*_-VLPs targeting H1 A/Idaho/07/2018 (Fig. S3d, e).

Antigen-specific CD4^+^ T cells were also measured in the BM, which serves as a major reservoir for long-term maintenance of memory CD4^+^ T cells following vaccination^29^. Immune cells were isolated from bilateral femurs 28 days post-boost and evaluated in parallel with splenocytes. Overall, cytokine signatures in the BM resembled those observed in the spleen. However, only the H1_*Y98F*_-VLP resulted in a significant increase in the frequency of H1-specific CD4^+^ T cells compared to the placebo group (*p* = 0.03) (Fig. 4c). The frequency of H1-specific CD4^+^ T cells in the BM strongly correlated with the frequency of responding cells in the spleen in mice vaccinated with H1_*Y98F*_-VLP (*r*_*s*_ = 1.00, *p* = 0.0167) but not H1_*WT*_-VLP (*r*_*s*_ = −0.1, *p* = 0.95), suggesting that a defect in recruitment contributes to the low frequency of H1-specific CD4^+^ T cells in the BM of H1_*WT*_-VLP-vaccinated mice (Fig. 4d). Similar to the spleen, the response to H1_*Y98F*_-VLP was dominated by IL-2^+^TNFα^+^IFNγ^−^ CD4^+^ T cells and IFNγ-expressing cells were virtually absent. Conversely, nearly 30% of BM CD4^+^ T cells elicited by H1_*WT*_-VLP were IFNγ^+^, and IL-2^+^TNFα^+^IFNγ^−^ cells were underrepresented in the BM compared to the spleen (24 vs. 32% of responding cells) (Fig. 4c).

Given that CD4^+^ T cells with the IL-2^+^TNFα^+^IFNγ^−^ phenotype have been shown to correlate with antibody responses to a number of vaccine antigens^30,31^, we sought to determine whether expansion of this population correlated with improved avidity maturation and functional antibody titers among mice vaccinated with H1_*Y98F*_-VLP. In the spleen, the frequency of IL-2^+^TNFα^+^IFNγ^−^ cells correlated with IgG avidity maturation in mice vaccinated with H1_*Y98F*_-VLP (*r*_*s*_ = 0.6809, *p* = 0.0355) but not H1_*WT*_ -VLP (*r*_*s*_ = 0.07976, *p* = 0.8355) (Fig. 4e). However, in the BM, this T cell population was strongly correlated with HI titers in both vaccine groups (*r*_*s*_ = 0.7957, *p* = 0.008) (Fig. 4f). This population also significantly correlated with MN titers (*r*_*s*_ = 0.7431, *p* = 0.0169) and IgG avidity (*r*_*s*_ = 0.8293, *p* = 0.0045), but not total IgG (*r*_*s*_ = 0.3598, *p* = 0.3054). Taken together, these findings suggest that enhanced recruitment of IL-2^+^TNFα^+^IFNγ^−^ CD4^+^ T cells to the BM may contribute to improved functional antibody responses in mice vaccinated with H1_*Y98F*_-VLP (Fig. 4f).

### H1_*Y98F*_-VLP results in reduced viral load and pulmonary inflammation following homologous challenge

**Fig. 5:**
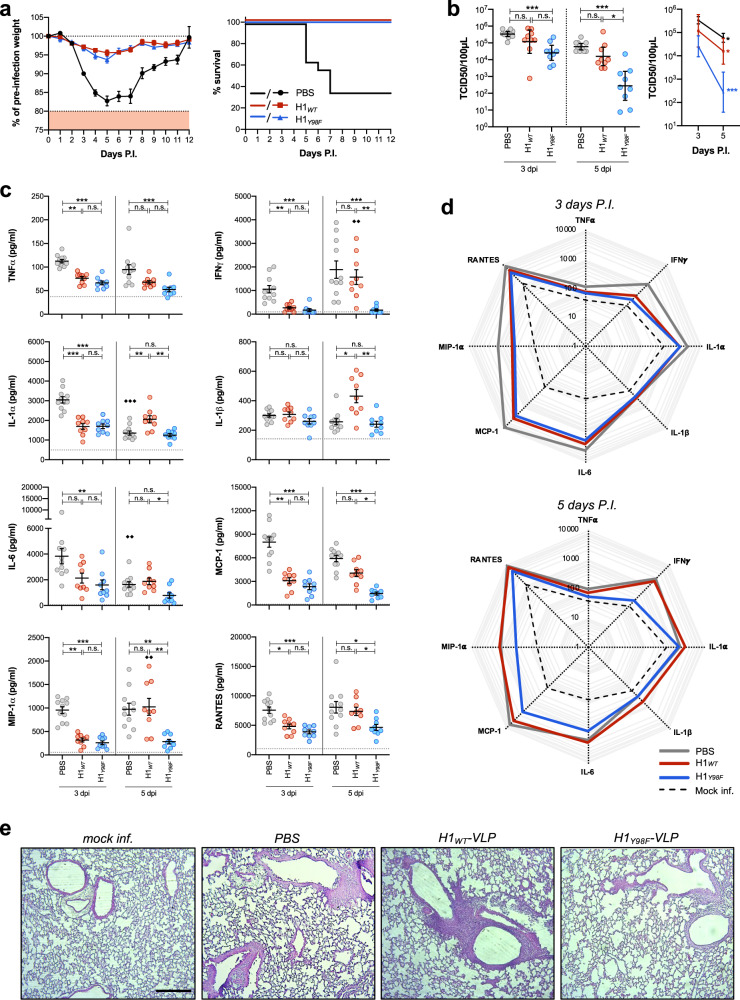
H1_*Y98F*_-VLP improves viral clearance and reduces pulmonary inflammation upon lethal challenge. Female Balb/c mice were challenged with 1.6 × 10^3^ TCID_50_ of H1N1 (A/California/07/09) 28 days post-vaccination with 3 µg H1_*WT*_*-* or H1_*Y98F*_-VLP or an equivalent volume of PBS. **a** Mean weight loss (left) and survival (right) were monitored for 12 dpi (*n* = 12–14/group, error bars represent SEM). Mice weighing <80% of their pre-challenge weight were euthanized. A subset of challenged mice (*n* = 9/group) were euthanized at 3 dpi and 5 dpi for evaluation of the viral load and pulmonary inflammation. **b** Viral titers in the supernatant of lung homogenates calculated using the Karber method and reported as TCID_50_/100 µl supernatant (GMT ± 95% CI). **c** Concentrations of cytokines and chemokines in the supernatant of lung homogenates measured by multiplex ELISA (mean ± SEM). The horizontal line represents the mean of mock-infected mice as a baseline. **d** Radar plots showing the cytokine profiles of mock-infected and infected lungs at 3 dpi and 5 dpi. **e** H&E stains of lungs collected 4 dpi (×10 magnification, scale bar = 100 µm). Statistical significance for **b** and **c** were determined by Kruskal–Wallis test with Dunn’s multiple comparisons. Comparisons between groups at the same time point are represented by * (*p < 0.033, **p < 0.01, ***p < 0.001) and comparisons within the same group over time are represented by ♦(^♦♦^*p* < 0.01, ^♦♦♦^*p* < 0.001).

In a study to compare protection following vaccination with H1_*WT*_- and H1_*Y98F*_-VLP, mice were challenged with 1.6 × 10^3^ times the median tissue culture infectious dose (TCID_50_) of H1N1 (A/California/07/09) 28 d post-vaccination with a single dose of 3 µg H1_*WT*_- or H1_*Y98F*_-VLP or an equivalent volume of PBS. Infection resulted in substantial weight loss (17.3 ± 1.3% at day 5) and 64.3% mortality in the placebo group. Consistent with previous reports^32^, vaccination with H1_*WT*_-VLP resulted in complete protection from lethal infection. Similarly, all mice vaccinated with H1_*Y98F*_-VLP survived and there was no significant difference in post-infection weight loss between the vaccinated groups (4–6% at day 5) (Fig. 5a). However, significant differences in the rate of viral clearance and pulmonary inflammation were observed.

To evaluate viral clearance, lungs were collected at 3 days post-infection (dpi) or 5 dpi and the TCID_50_ of lung homogenates was determined (Fig. 5b). Vaccination with H1_*Y98F*_-VLP, but not H1_*WT*_-VLP, resulted in a decrease in the viral titer compared to placebo at 3 dpi (*p* < 0.001). In both groups, viral titers were inversely correlated with HI and MN titers (HI *r*_*s*_ = −0.695, *p* = 0.001; MN *r*_*s*_ = −0.7067, *p* = 0.001). By 5 dpi, viral titers in mice vaccinated with H1_*Y98F*_-VLP were nearly 2-log lower than mice vaccinated with H1_*WT*_-VLP (*p* = 0.03) and there was no significant difference between the placebo and H1_*WT*_-VLP-vaccinated mice (*p* = 0.31).

Lung homogenates were evaluated by multiplex ELISA (Quansys) to quantify several cytokines (TNFα, IFNγ, IL-1α, IL-1β and IL-6) and chemokines (MCP-1, MIP-1α and RANTES) implicated in influenza-mediated acute lung injury (Fig. 5c, d)^33,34^. Baseline levels were established using lung homogenates from mock-infected mice. Overall, vaccination with either H1_*WT*_-VLP or H1_*Y98F*_-VLP resulted in reduced cytokines and chemokines compared to the placebo group at 3 dpi, but there were no significant differences between the vaccinated groups. However, by 5 dpi there was a marked divergence in pulmonary inflammation and all cytokine/chemokine levels except for TNFα were significantly lower in the H1_*Y98F*_-VLP vaccinated mice compared to the H1_*WT*_-VLP group. Strikingly, mice vaccinated with H1_*WT*_-VLP exhibited a significant increase in IFNγ compared to 3 dpi (*p* = 0.005) with levels nearing the placebo group (1570 ± 315 and 1887 ± 367 pg/ml, respectively). IFNγ levels correlated with the viral titer (*r*_*s*_ = −0.7050, *p* = 0.001) in both vaccinated groups and remained near the baseline in mice that received H1_*Y98F*_-VLP (172 ± 46 pg/ml). IL-1β, MIP-1α and RANTES levels followed similar trends. Consistent with these findings, histopathological evaluation of lungs collected at 4 dpi revealed that mice vaccinated with H1_*Y98F*_-VLP had less pulmonary inflammation compared to H1_*WT*_-VLP-vaccinated mice and more closely resembled mock-infected animals (Fig. 5e). Together, these findings suggest that while both vaccines protected from lethal influenza infection, mice vaccinated with H1_*Y98F*_-VLP were better able to control and clear the virus and exhibited reduced influenza-associated lung inflammation.

## Discussion

Vaccination is the most effective strategy to prevent influenza-associated morbidity and mortality, however, vaccine effectiveness is often limited by variable immunogenicity and rapidly waning protection^35,36^. We have demonstrated that SA binding properties of influenza HA influence vaccine responses and that ablation of HA-SA interactions can improve the quality and durability of immune responses in mice. Non-binding H1 mutants were generated using the previously described Y98F mutation, which prevents SA binding without affecting antibody recognition of the globular head or RBD^17^. Although historically used as probes to identify HA-specific B cells^17,23,37^ and more recently in studies targeting the generation of stem-specific antibodies^38,39^, the Y98F mutation was not thought to influence immunogenicity. By directly comparing H1_*WT*_ and H1_*Y98F*_ antigens in vaccination and challenge models, we have demonstrated that HA-SA binding can have significant impact on influenza vaccine responses.

Antibodies that mediate HI have long been shown to provide protection and are widely considered to be a good predictor of vaccine efficacy. Thus, the induction of high HI titers is important for licensure of influenza vaccines in many jurisdictions^40–42^. MN titers also correlate with protection and provide useful insight into the functional neutralizing capacity of vaccine-induced antibodies^43^. In this study we demonstrated that ablation of HA-SA binding has broad impact on the humoral response to vaccination. Consistent with previous reports, we observed a strong correlation between HI and MN titers^43^ and pre-infection antibody were inversely correlated with pulmonary peak viral load (3 dpi) in both vaccine groups, suggesting that these antibodies play a direct role in controlling infection. While viral titers in the lungs do not always correlate with morbidity^44,45^, the strong inverse correlations between viral titers and pulmonary inflammatory cytokines and infiltrates suggest that the viral load is a good reflection of disease severity in this model. The improved functional antibody response elicited by H1_*Y98F*_-VLP is therefore likely to have played an important role in reducing the severity of infection.

Our data suggest that eliminating HA-SA binding may also improve the durability of the antibody response to influenza vaccines. Vaccine-induced HI titers typically decline rapidly post-vaccination^46–49^, consistent with our observations in mice that received H1_*WT*_-VLP. In sharp contrast, HI and MN titers in mice vaccinated with H1_*Y98F*_-VLP were maintained at peak levels until 7 mpv. While the mechanisms underlying this observation are under further investigation, improved specificity and longevity of the GC reaction and an increase in HA-specific PC in the BM likely play an important role. BM PC can persist for decades following antigen exposure in humans and are thought to mediate long-term maintenance of serum antibody titers following vaccination^18,50^. Our finding that increased frequencies of BM PC correlate with HI, MN, and total IgG titers at 7 mpv suggests that this population is central to the durability of humoral response elicited by H1_*Y98F*_-VLP. Weisel et al. have shown that long-lived PC in the BM arise from late GC B cells (18–20 days) after a prolonged period of somatic hypermutation and avidity maturation^51^. The increased HA-specific BM PC we found in the H1_*Y98F*_-VLP group is therefore likely a reflection of the increased HA-specific B cells and T_FH_ cells in the GC of these animals at 19 dpv.

Although influenza vaccine developers have focused primarily on humoral responses, antigen-specific T cells undoubtedly play important roles in protection and often generate more broad and cross-reactive responses than antibodies^52^. CD4^+^ T cells support both B cell affinity maturation and CD8^+^ T cell responses and can mediate protection in a murine vaccination/challenge model in the absence of neutralizing antibodies^53^. Previous studies have demonstrated that plant-based H1_*WT*_-VLPs elicit strong CD4^+^ T cell responses in both mice and humans with increased CD4^+^ T cell polyfunctionality compared to inactivated vaccines^32,54–57^. The overall pattern of CD4^+^ T cell responses in this study was consistent with previous reports and was not compromised in the absence of HA-SA interactions. However, there were no differences between vaccine groups after a single dose, suggesting that enhanced viral clearance in this model was driven primarily by the better humoral response to the H1_*Y98F*_-VLP vaccine. Interestingly, two doses of H1_*Y98F*_-VLP resulted in significant expansion of the IL-2^+^TNFα^+^IFNγ^−^ population, which was strongly correlated with humoral responses. Others have demonstrated that this population is comprised of Th1 cells and a population of primed but uncommitted T helper cells (Thpp) with high proliferative and differentiation potential^58^. Thpp cells are frequently generated in primary responses to antigens including influenza^58^, thus, it is not surprising that both vaccines elicited similar frequencies of IL-2^+^TNFα^+^IFNγ^−^ CD4^+^ T cells after a single dose. However, subsequent exposure to influenza HA typically results in expression of IFNγ^58,59^. Expansion of the IFNγ^−^ population in the spleen upon boosting with H1_*Y98F*_-VLP but not the H1_*WT*_-VLP is reminiscent of the CD4^+^ T cell responses to protein vaccines such as hepatitis B, diphtheria and tetanus, that are dominated by Thpp cells and elicit robust and durable antibody responses^30,31,60^. The Thpp bias induced by the “non-binding” HA and concomitantly higher HI titers were also observed with H1 A/Idaho/07/2018, suggesting that the impact on CD4^+^ T cells could broadly influence H1 humoral responses.

To our knowledge, Thpp cells have not been described in the BM and it is unknown whether this population is a common feature of other protein vaccines. However, BM CD4^+^ T cells are a major reservoir for long-lasting immunity and are known to provide efficient help to B cells^29,61^. Thus, enhanced homing of CD4^+^ T cells to the BM may contribute to the enhanced durability of the responses we observed following H1_*Y98F*_-VLP vaccination. Interestingly, IL-2^+^TNFα^+^IFNγ^−^ CD4^+^ T cells in the BM significantly correlated with HI titers, MN titers and IgG avidity, but not total IgG titers. One possible explanation for this discrepancy is that the IL-2^+^TNFα^+^IFNγ^−^ CD4^+^ T cells play a role in B cell selection, giving rise to antibodies with increased avidity and functional capacity.

Beyond the possibility that Y98F HAs behave similarly to traditional protein vaccines, the mechanisms underlying their improved immunogenicity are not yet clear. However, a likely contributor is better trafficking to the draining LN in the absence of SA binding. Terminal α2,3 and α2,6 SAs are widely distributed throughout the body, including on skeletal muscle cells^12,62,63^. Thus, the H1_*WT*_-VLP and recombinant WT HAs may be more likely to be sequestered at the site of injection or en route to the draining LN due to HA-SA interactions. In support this hypothesis, previous work has demonstrated that while eGFP-labeled H5-VLP can be detected in the popliteal LN 10 min after footpad injection^64^, a substantial eGFP signal remains at the site of injection for up to 24 h. Soluble eGFP was not retained in the footpad, suggesting that SA binding may contribute to injection site retention^65^. Although depot-type adjuvants that “trickle” antigen into the draining LN can enhance affinity maturation through prolonged antigen availability in the GC^66^, the rapid decline in T_FH_ cells suggests that this is not occurring in mice vaccinated with H1_*WT*_-VLP. It is possible that H1_*WT*_-VLPs retained at the injection site are degraded prior to delivery to the draining LN. Thus, retention of H1_*WT*_ antigen at the site of injection may reduce the concentration of antigen delivered to draining LN without providing sustained antigen presentation. In addition, HA-SA interactions may limit immune recognition of important neutralizing epitopes on the globular head of HA through steric hindrance. Ongoing investigations aim to characterize the impact of SA binding on trafficking of the H1 antigen to the draining LN and epitope specificity of the immune response.

In addition to these mechanistic studies, we are also evaluating the generalizability of these findings with respect to different influenza strain HAs and different production platforms. The fact that a H1_*Y98F*_-VLP targeting an antigenically distinct H1 (A/Idaho/07/2018) has similar immune effects suggests that eliminating HA-SA interactions may be a promising strategy to improve immunogenicity of vaccines against current and emerging strains of influenza. Confirmatory studies with additional non-binding VLPs (e.g.: H3N2 and B strains) will be required to evaluate the broad generalizability of this approach. The fact that improved humoral responses were observed with soluble H1_*Y98F*_ trimers (A/Brisbane/02/2018) suggests that this strategy may confer similar benefits across recombinant vaccine platforms. Although egg-based propagation of influenza A strains bearing Y98F HA has been described^16^, mutations that restore HA binding may limit the viability of this approach in platforms requiring the growth of live virus^67^. Finally, since receptor-binding proteins are often targets of choice for viral vaccines and many of the receptors for these viruses have wide tissue distribution, our observations raise the possibility that manipulation of non-cognate binding interactions may have application beyond influenza vaccines. For example, SA binding is a common feature of many viruses and vaccine antigens including the hemagglutinin-neuraminidase protein of mumps virus^68^, the VP8* domain of bovine-human reassortant rotavirus vaccine strains^69^, and SARS-CoV-2 spike protein^70^.

Taken together, we have demonstrated that altering the binding of HA to its SA receptor can have a significant impact on influenza vaccine-mediated immunity and viral clearance in mice. Elimination of the HA-SA interaction may be a simple, effective and readily implemented strategy to improve both the quality and durability of influenza vaccine responses. Although there is still much to learn mechanistically, if these findings are confirmed in human studies, the use of non-binding HAs may make an important contribution to the development of next-generation influenza vaccines.

## Methods

### H1_*WT*_ and H1_*Y98F*_ expression cassettes

The sequences encoding mature WT and Y98F HA0 A/California/07/2009 fused to alfalfa PDI secretion signal peptide (PDISP) were cloned into 2X35S/CPMV160/NOS expression system using PCR-based methods. To generate the H1_*WT*_ expression cassette, the PDISP-A/California/07/2009 coding sequence was amplified using primers IF-CPMV(fl5′UTR)_SpPDI.c (5′-TCGTGCTTCGGCACCAGTACAATGGCGAAAAACGT-TGCGATTTTCGGCT-3′) and IF-H1cTMCT.S1-4r (5′-ACTAAAGAAAATAGGCCTTTA-AATACATATTCTACACTGTAGAGAC-3′). To generate the H1_*Y98F*_ expression cassette, the PDISP-A/California/07/2009 coding sequence with the mutated Y98F amino acid (H3 numbering) was amplified first using primers IF-CPMV(fl5′UTR)_SpPDI.c and H1_Cal(Y91F).r (5′-AAATCTCCTGGGAAACACGTTCCATTGTCTGAACTAGGTGTT-TCCACAA-3′), and second using primers H1_Cal(Y91F).c (5′-AGACAATGGAACGTGT-TTCCCAGGAGATTTCATCGATTATGAGGAGCTA-3′) and IF-H1cTMCT.S14r. The PCR products from both amplifications were mixed and used as a template for amplification using primers IF-CPMV(fl5′UTR)_SpPDI.c and IF-H1cTMCT.S14r. The final amplification products were assembled into the pCAMBIA binary plasmid containing 2X35S/CPMV160/NOS and linearized by digestion with *Sac*II and *Stu*I restriction enzymes using the In-Fusion cloning system (Clontech, Mountain View, CA).

### Protein expression and VLP purification

VLPs were produced by transient transfection of *Nicotiana benthamiana* plants with *Agrobacterium tumefaciens* carrying H1_*WT*_ or H1_*Y98F*_ expression cassettes. Briefly, *N. benthamiana* plants (41–44 days old) were vacuum infiltrated in batches and the aerial parts of the plants were harvested and frozen (−80 °C) after 7 days of incubation. To extract and purify VLPs, frozen plant leaves were homogenized in 1 l of extraction buffer [50 mM Tris, 500 mM NaCl (pH 7.4) with 0.04% (w/v) Na_2_S_2_O_5_]/kg biomass. The homogenate was pressed through a 400 µm nylon filter and the fluid was retained. Filtrates were clarified by centrifugation (5000 × *g)* and filtration (1.2 µm glass fiber, 3 M Zeta Plus, 0.45-0.2 µm filter) and then concentrated by centrifugation (75,000 × *g*, 20 min). VLPs were further concentrated and purified by ultracentrifugation over an iodixanol density gradient (120,000 × *g*, 2 h). VLP-rich fractions were pooled and dialyzed against 50 mM NaPO4, 65 mM NaCl, 0.005% Tween 80 (pH 6.0). This clarified extract was captured on a Poros HS column (Thermo Scientific) equilibrated in 50 mM NaPO_4_, 1 M NaCl, 0.005% Tween 80. After washing with 25 mM Tris, 0.005% Tween 80 (pH 8.0), the VLPs were eluted with 50 mM NaPO_4_, 700 mM NaCl, 0.005% Tween 80 (pH 6.0). Purified VLPs were dialyzed against formulation buffer (100 mM NaKPO_4_, 150 mM NaCl, 0.01% Tween 80 (pH 7.4)) and passed through a 0.22 µm filter for sterilization. Protein concentrations were determined using Pierce^TM^ micro BCA protein assay kit according to the manufacturer’s instructions.

### Gel electrophoresis and immunoblot analysis

To confirm HA expression, 2 g of biomass were homogenized in 4 ml extraction buffer with 1% phenylmethylsulfonyl fluoride. Homogenates were clarified by centrifugation (10,000 × *g*, 10 min) and the crude extracts (25 µl/sample) were separated a Criterion XT 4–12% Bis-Tris gel (Bio-Rad) under reducing conditions and then transferred onto a PVDF membrane. Successful transfer was confirmed using ponceau red staining followed by de-staining with water. Membranes were blocked overnight (4 °C) with 5% skim milk in TBST (tris-buffered saline, 0.1% Tween 20) and then incubated with rabbit polyclonal anti-H1 (Cat. No. IT-003-SW, Immune Technology) diluted 1:500 in TBST + 2% skim milk for 1 h at room temperature (RT). Membranes were then incubated for 1 h (RT) with horseradish peroxidase (HRP)-conjugated goat anti-rabbit IgG (Cat. No. IT-200-01, Immune Technology) diluted 1:20,000 in TBST + 2% skim milk. Bands were developed using Super Signal West Pico chemiluminescent substrate (Thermo Fisher) and detected on X-ray films. For VLP composition and purity analysis, purified VLP products (5 µg/sample) were separated on a 4–12% Bis-Tris gel as described above followed by staining with biosafe Coomassie G-250 (Bio-Rad). Gels were imaged using ChemiDoc^TM^ XRS + system (Bio-Rad). See Supplementary Fig. 11 for unmodified gel and blot images.

### Transmission electron microscopy (TEM)

VLP samples were diluted to 100 µg/ml in PBS and 5 µl of each were placed on 200 copper grids (Agar Scientific) for 45 s. Grids were washed 2x (1 min each) with 5 µl distilled water followed by two incubations with 1.5% uranyl acetate. Excess fluid was removed and samples were left to air dry. Grids were imaged on a Tecnai G2 Spirit Twin 120 kV Cryo-TEM (FEI) equipped with a Gatan Ultrascan 4000 CCD camera model 895 (Gatan).

### Surface plasmon resonance

Binding of VLP to biotinylated ɑ-2,6 sialic acid glycans (6′-sialyl(LacNAc)-PEG-biotin) was quantified by SPR using a Biacore^TM^ 8 K system (Cytiva, formerly GE Healthcare Life Sciences). Biotinylated synthetic glycan (Sussex Research Laboratories Inc.) was immobilized to a Series S sensor chip SA at a minimum target of 400 resonance units (RU) in the test flow cells. VLPs were diluted in HBS-EP + Buffer (assay running buffer) and injected at a flow rate of 50 μl/min (120 s contact time) at 4 °C. H1_*WT*_-VLP was diluted 150x and 100x (2.5 and 4 µg/ml) and H1_*Y98F*_-VLP was diluted 10x and 5x (88 and 176 µg/ml). The standard curve included 8 dilutions ranging from 10 to 0.08 μg/ml. The flow was initially directed over a mock surface to which no protein is bound, followed by the biotinylated glycan. Response from the protein surface is corrected for the response from the mock surface (surface reference). Data analysis was performed using Biacore^TM^ 8 K Insight Evaluation Software (version 2.0.15.12933) using concentration analysis mode (fitting function linear analysis). The slope of the association curve was measured for each VLP and normalized for HA content. Relative binding was calculated based on the adjusted slopes, with the H1_*Y98F*_-VLP assigned a value of 100%.

### Vaccination and sample collection

All animal procedures were carried out in accordance with guidelines of the Canadian Council on Animal Care, as approved by the Animal Care Committee of McGill University. Female Balb/c mice (8–10 weeks old, Charles River Laboratories) were immunized by injection into the quadricep muscle with VLP formulations containing 0.5–3 µg HA (50 µl total in PBS). Mice were vaccinated on day 0 and boosted on day 21 (when indicated). To evaluate humoral and cell-mediated immune responses mice were euthanized on day 28 (one-dose) or day 49 (28 days post-boost) by CO_2_ asphyxiation. A small group of mice were maintained for 7 months to evaluate long-term immunity and sera were collected monthly.

Blood was collected from the left lateral saphenous vein before each vaccination and by cardiac puncture at study endpoints. Sera were obtained by centrifugation of blood in microtainer serum separator tubes (Beckton Dickinson) (8000 × *g*, 10 min) and stored at −20 °C until further analysis. Spleens were collected in Hank’s balanced salt solution (HBSS) without calcium and magnesium (Wisent). Single cell suspensions were prepared by passing spleens through a 70 µm strainer (BD Biosciences) and RBC were lysed using ACK buffer (0.15 M NH_4_Cl, 1 mM KHCO_3_, 0.1 mM NA_2_EDTA, pH 7.4). Cells were washed in HBSS and then resuspended in RPMI (Wisent) supplemented with 10% fetal bovine serum (Wisent), 1 mM penicillin/streptomycin (Wisent) and 0.5 mM β-mercaptoethanol (Sigma) (complete RPMI, cRPMI). Femurs were collected in cRPMI, sterilized in 70% ethanol (2 min) and rinsed with HBSS. Femurs were cut at one end and BM was extracted by centrifugation (8000 RPM, 30 s). Single cell suspensions were obtained as described for spleens.

To evaluate GC responses mice were vaccinated in the right hind limb footpad with 0.5 µg VLP (30 µl total in PBS). Draining popliteal LN were collected at indicated time points and digested with collagenase D (1 mg/ml; Sigma) and DNaseI (10 µg/ml; Sigma) (40 min, 37 °C, shaking at 220 RPM) prior to mechanical dissociation as described for spleens.

### Antibody titer measurement

Total HA-specific IgG was quantified by enzyme-linked immunosorbent assay (ELISA). U-bottom, high-binding 96-well plates (Greiner) were coated overnight (4 °C) with 2 µg/ml recombinant HA (Immune Technologies) or HA_*WT*_-VLP (Medicago Inc.) diluted in 100 mM bicarbonate/carbonate buffer at pH 9.6 (50 µl/well). For the standard curve, wells were coated with two-fold dilutions of purified mouse IgG (Sigma) starting at 2000 ng/ml. Wells were blocked with 2% bovine serum albumin (Sigma) in PBS with 0.05% TWEEN 20 (Fisher Scientific) (150 µl/well). Heat-inactivated sera diluted 1:50 in blocking buffer were added to plates in duplicate (50 µl/well) and incubated for 1 h at 37 °C. HA-specific IgG was detected using HRP-conjugated anti-mouse IgG (Cat. No. 1036-95; Southern Biotech) diluted 1:20,000 in blocking buffer (75 µl/well, 30 min, 37 °C) followed by incubation with 3,3′,5,5′-Tetramethyl benzidine substrate (Millipore) for 15 min (RT). Development was stopped after 15 min using 0.5 M H_2_SO_4_ (50 µl/well) and the optical density was measured at 450 nm (EL800 microplate reader; BioTek Instruments Inc.). To evaluate the avidity of HA-specific IgG, wells containing bound antibody were incubated with urea (0M–8M) for 15 min and re-blocked for 1 h prior to detection. Avidity index (AI) = [IgG titer 2–8 M urea/IgG titer 0 M urea].

To measure antibodies that mediate HI sera were diluted 1:4 in receptor-destroying enzyme (RDE; Denka Seiken) and incubated 18–20 h at 37 °C. Sera were inactivated for 30 min at 56 °C then further diluted to 1:10 with PBS. Two-fold serial dilutions of RDE-inactivated sera were added to a V-well microtiter plate (25 µl/well) and incubated for 30 min with 4 HA units of virus (25 µl/well) at RT. 50 µl of 0.5% turkey RBCs was added to each well and incubated 30 min at RT. Wells were examined visually for RBC teardrop formation upon plate tilting, indicating inhibition of hemagglutination^25^. Titers are reported as the reciprocal of the highest dilution to inhibit hemagglutination. Titers below the limit of detection (<10) were assigned a value of 5 for statistical analysis.

To measure antibodies that mediate MN MDCK cells (ATCC CCL-34) were seeded into flat-bottom 96-well plates (50,000/well) in HyClone SFM4MegaVir medium (Cytiva) supplemented with 10 µg/ml gentamicin (Gibco Life Technologies), 100,000 U/ml penicillin G (Gibco Life Technologies) and 20 µg/ml glutamine (Wisent). Two-fold serial dilutions of heat-inactivated sera (56 °C, 30 min) were incubated with 100 TCID_50_ of virus for 2 h (37 °C, 5% CO_2_) and then added to the MDCK monolayers with 1x TPCK (tolylsulfonyl phenylalanyl chloromethyl ketone)-treated trypsin. Plates were incubated for 3 h (37 °C, 5% CO_2_) and then medium was removed from each well and replaced with fresh MegaVir with 1x TPCK-treated trypsin. Cells were observed for cytopathic effects (CPE) for 3–5 days and the MN titer was defined as the reciprocal of the highest dilution to prevent CPE. Titers below the limit of detection (<10) were assigned a value of 5 for statistical analysis.

### ELISpot

HA-specific IgG antibody secreting cells (ASC) were quantified by ELISpot (Mouse IgG ELISpot^BASIC^, Mabtech). Sterile PVDF membrane plates (Millipore) were coated with Anti-IgG capture antibody and blocked according to the manufacturer’s guidelines. To quantify in vivo activated ASCs, wells were seeded with 250,000 (BM) or 500,000 (splenocyte) freshly isolated cells and incubated at 37 °C, 5% CO_2_ for 16–24 h. HA-specific ASCs were detected according to the manufacturer’s guidelines using 1 µg/ml biotinylated HA (immune tech, biotinylated using Sulfo-NHS-LC-Biotin). To evaluate memory ASCs, freshly isolated cells were polyclonally activated with 0.5 µg/ml R848 and 2.5 ng/ml recombinant mouse IL-2 (1.5 × 10^6^ cells/ml in 24-well plates) for 72 h (37 °C, 5% CO_2_). Activated cells were re-counted and the assay was carried out as described above.

### Flow cytometry

To identify total GC B cells and T_FH_ cells, freshly isolated cells from the popliteal LN (1 × 10^6^ cells/200 µl) were washed 1x with PBS in a 96-well round-bottom plate (350 × *g*, 7 min, 4 °C) and labeled with Fixable Viability Dye eFluor 780 (eBioscience) (20 min, 4 °C). Cells were washed 3x followed by incubation with Fc Block (1 µl/sample; BD Biosciences Cat. No. 553142) for 15 min at 4 °C. Samples were incubated for an additional 30 min upon addition of the surface cocktail containing the following anti-mouse antibodies: anti-CD3 Alexa Fluor 700 (1 µl/sample; Biolegend Cat. No. 100216), anti-CD4 V500 (1 µl/sample; BD Cat. No. 560782), anti-CD19 PE-CF594 (1 µl/sample; BD Cat. No. 562291), anti-Fas PE (2.5 µl/sample; Thermo Fisher Cat. No. 12-0951-83), anti-GL7 FITC or Alexa Fluor 647 (0.5 µl/sample; Biolegend Cat. No. 144607/144606), anti-PD-1 Alexa Fluor 647 (0.25 µl/sample; Biolegend Cat. No. 135230), anti-CXCR5 biotin (2 µl/sample; BD Cat. No. 551960). Cells were washed 2x followed by incubation with streptavidin BV421 (BD Biosciences) for 40 min at 4 °C. Cells were washed 3x and fixed for 30 min (Fix/Perm solution, BD Biosciences) prior to acquisition. To quantify H1-specific GC B cells, cell suspensions were incubated with 1 µg/ml H1_*Y98F*_-VLP for 1 h prior to surface staining and VLP-bound cells were detected using anti-H1 FITC (1 µl/sample; Immune Technologies Cat. No. IT-003-001M5-FITC) using the above panel omitting anti-CXCR5 and anti-PD-1. For the full gating strategy, see Supplementary Fig. 5.

To identify antigen-specific CD4^+^ T cells, freshly isolated splenocyte or BM immune cell suspensions (1 × 10^6^/200 µl in a 96-well U-bottom plate) were stimulated with 10% cRPMI (negative control), 2–2.5 µg/ml homologous HA_*WT*_-VLP (18 h), or a pool of overlapping peptides (15aa) spanning the HA sequence (6 h, BEI resources) (37 °C, 5% CO_2_). Golgi Stop and Golgi Plug (BD Biosciences) were added 5 h before the end of the stimulation according to the manufacturer’s instructions. Cells were washed 2x with PBS (320 × *g*, 8 min, 4 °C) and labeled with Fixable Viability Dye eFluor 780 (eBioscience) (20 min, 4 °C). Cells were washed 3x followed by incubation with Fc Block (BD Biosciences) for 15 min at 4 °C. Samples were incubated for an additional 30 min upon addition of the surface cocktail containing the following antibodies: anti-CD3 FITC (1 µl/sample; Thermo Fisher Cat. No. 11-0031-86), anti-CD4 V500 (1 µl/sample; BD Cat. No. 560782) anti-CD8 PerCP-Cy5.5 (1 µl/sample; BD Cat. No. 551162), anti-CD44 BUV395 (1 µl/sample; BD Cat. No. 740215) and anti-CD62L BUV373 (0.5 µl/sample; BD Cat. No. 612-833). Cells were washed 3x and fixed (Fix/Perm solution, BD Biosciences) overnight. For detection of intracellular cytokines, fixed cells were washed 3x in perm/wash buffer (BD Biosciences) followed by intracellular staining with the following antibodies (30 min, 4 °C): anti-IL-2 APC (1 µl/sample; Biolegend Cat. No. 503810), anti-IFNγ PE (1 µl/sample; BD Cat. No. 562020) and anti-TNFα eFluor450 (1 µl/sample; Invitrogen Cat. No. 48-7321-82). Cells were washed 3x in perm/wash buffer and then resuspended in PBS for acquisition. For the full gating strategy, see Supplementary Fig. 8.

All flow cytometry was conducted using a BD LSRFortessa or BD LSRFortessa X20 cell analyzer. Data was analyzed using FlowJo software (Treestar, Ashland).

### Challenge

Mice were challenged with 1.6 × 10^3^ times the median tissue culture infectious dose (TCID_50_) of H1N1 A/California/07/09 (National Microbiology Laboratory, Public Health Agency of Canada) diluted in HyClone SFM4MegaVir (Cytiva) supplemented with 10 µg/ml gentamicin (Gibco Life Technologies), 100,000 U/ml penicillin G (Sigma) and 20 µg/ml glutamine (Wisent). Mice were anesthetized using isoflurane and infected by intranasal instillation (25 µl/nare). Mice were monitored for weight loss for 12 days post-infection and were euthanized if they lost ≥20% of their pre-infection weight (humane end-point). A subset of mice in each group was sacrificed 3–5 days post-infection (dpi) and lungs were harvested for evaluation of viral load and inflammation.

### Preparation of lung homogenates

Lungs were individually homogenized in an equal amount (wt/wt) of complete MegaVir (see above) using micro-tube homogenizer for 3 min. Solid tissue was removed by centrifugation (14,000 × *g*, 5 min, 4 °C) and supernatants were stored at −80 °C until further analysis.

### Lung viral load

Infectious virus titers in the supernatant of lung homogenates were determined by TCID_50_. MDCK monolayers were prepared in 96-well plates as described above. Lung homogenates were serially diluted (1:10) in complete MegaVir media (see above) supplemented with 2 mg/ml TPCK-treated trypsin (Sigma) and applied to MDCK monolayers for 1 h (37 °C, 5% CO_2_). Diluted homogenates were then removed from the wells and replaced with fresh MegaVir-trypsin. Cells were incubated for 4 days (37 °C, 5% CO_2_) and then evaluated for CPE. The TCID_50_ was calculated using the Karber method^71^.

### Multiplex ELISA

Pulmonary cytokines in lung homogenates obtained from infected and non-infected mice were measured using the Q-Plex^TM^ mouse cytokine screen (16-plex, Quansys Bio) according to the manufacturer’s instructions.

### Histology

Whole lungs were inflated with 10% formalin (Fisher Scientific) and then fixed for ≥24 h in 10% formalin. Fixed lungs were embedded in paraffin (Leica), cut into 4 µm sections, and applied to glass slides. Slides were washed with xylene (Chaptec) and then immersed in ethanol for 10 min. Slides were rinsed with distilled water and then briefly submerged in 50% Harris hematoxylin (HHS-32; Sigma) diluted in distilled water. Slides were rinsed under running tap water, washed 10x with ethanol, and then stained with eosin-phloxin B [100 ml 1% eosin Y (Sigma), 10 ml phloxin B (Sigma), 780 ml ethanol, 4 ml glacial acetic acid (Fisher Scientific)]. Slides were immersed in ethanol for 10 min, dried, and then submerged in xylene for 10 min. Slides were fixed with two drops of acrytol (Leica) with a coverslip. Sections were stained with hematoxylin and eosin as previously described^32^. Images were obtained using a Zeiss Primo Star light microscope equipped with an AxioCam ERc5s (Zeiss) camera.

### Reporting summary

Further information on research design is available in the Nature Research Reporting Summary linked to this article.

## Supplementary information

Supplemental Material

REPORTING SUMMARY

## Data Availability

Data generated in the current study are available from the corresponding author.
