# Supplementary material for: Elimination of receptor binding by influenza hemagglutinin improves vaccine-induced immunity
Source: NPJ Vaccines. 2022 Apr 11;7:42. doi: 10.1038/s41541-022-00463-3 (PMC9001741; doi:10.1038/s41541-022-00463-3)
Supplement: Supplementary file 1 — Supplemental Material [file 41541_2022_463_MOESM1_ESM.pdf]

SUPPLEMENTAL FIGURES AND LEGENDS

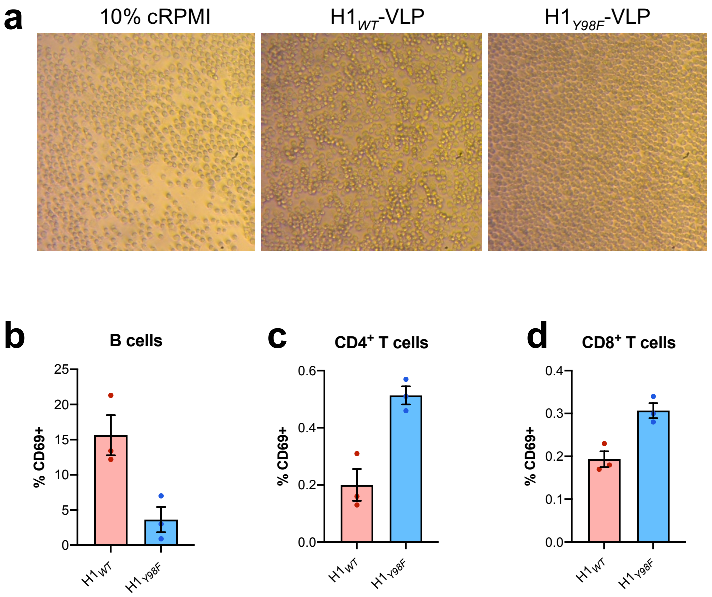

Supplementary figure 1. Elimination of SA binding prevents PBMC clustering and impacts downstream cell activation. (a) $1x10^6$ Human PBMC were co-incubated with H1$_{WT}$- or H1$_{Y98F}$-VLP (5µg/mL) for 30min (37°C, 5%CO_2). Cells were visualized using a Zeiss Primovert inverted microscope equipped with an AxioCam ERc5s camera (10X). To evaluate activation $1x10^6$ Human PBMC were co-incubated with H1$_{WT}$- or H1$_{Y98F}$-VLP (1µg/mL) for 6h (37°C, 5%CO_2) and CD69 expression was measured in (b) B cells, (c) $CD4^+$ T cells, and (d) $CD8^+$ T cells by flow cytometry. Background values obtained from non-stimulated samples were subtracted from values obtained following stimulation with VLP.

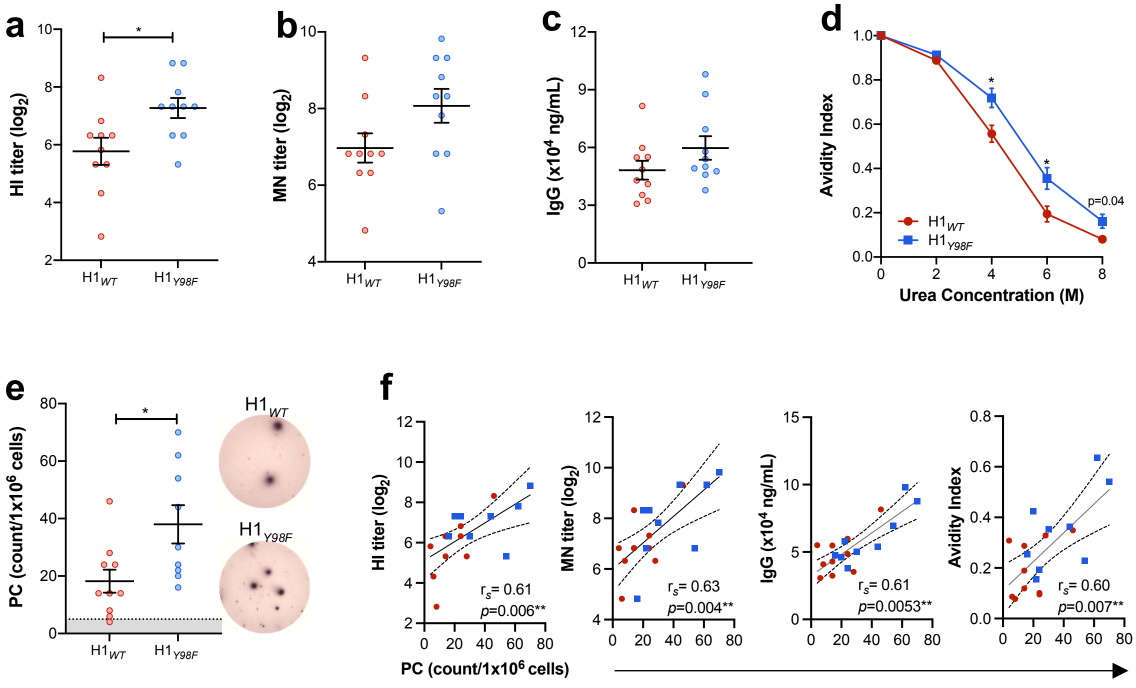

Supplementary figure 2. Improved humoral immunity elicited by H1$_{Y98F}$-VLP is maintained post-boost. Female Balb/c mice were vaccinated with 0.5µg H1$_{WT}$- or H1$_{Y98F}$-VLP on days 0 and 21. Mice were euthanized 28d post-boost and sera were collected to evaluate (a) Total H1-specific IgG titers by ELISA, (b) HI titers, (c) MN titers, and (d) IgG avidity indices. Immune cells were also isolated from the BM to evaluate (e) the frequency of H1-specific IgG producing PC. (f) Spearman's rank correlation technique was applied to evaluate the relationship between the frequency of PC and HI titers, MN titers, IgG titers and IgG avidity indices following incubation with 6M Urea. For (a-c) and (e) statistical significance was evaluated by Mann-Whitney test and for (d) statistical significance was evaluated by Mann-Whitney test at each concentration of Urea with Holm-Sidak's multiple comparisons test (*p<0.033).

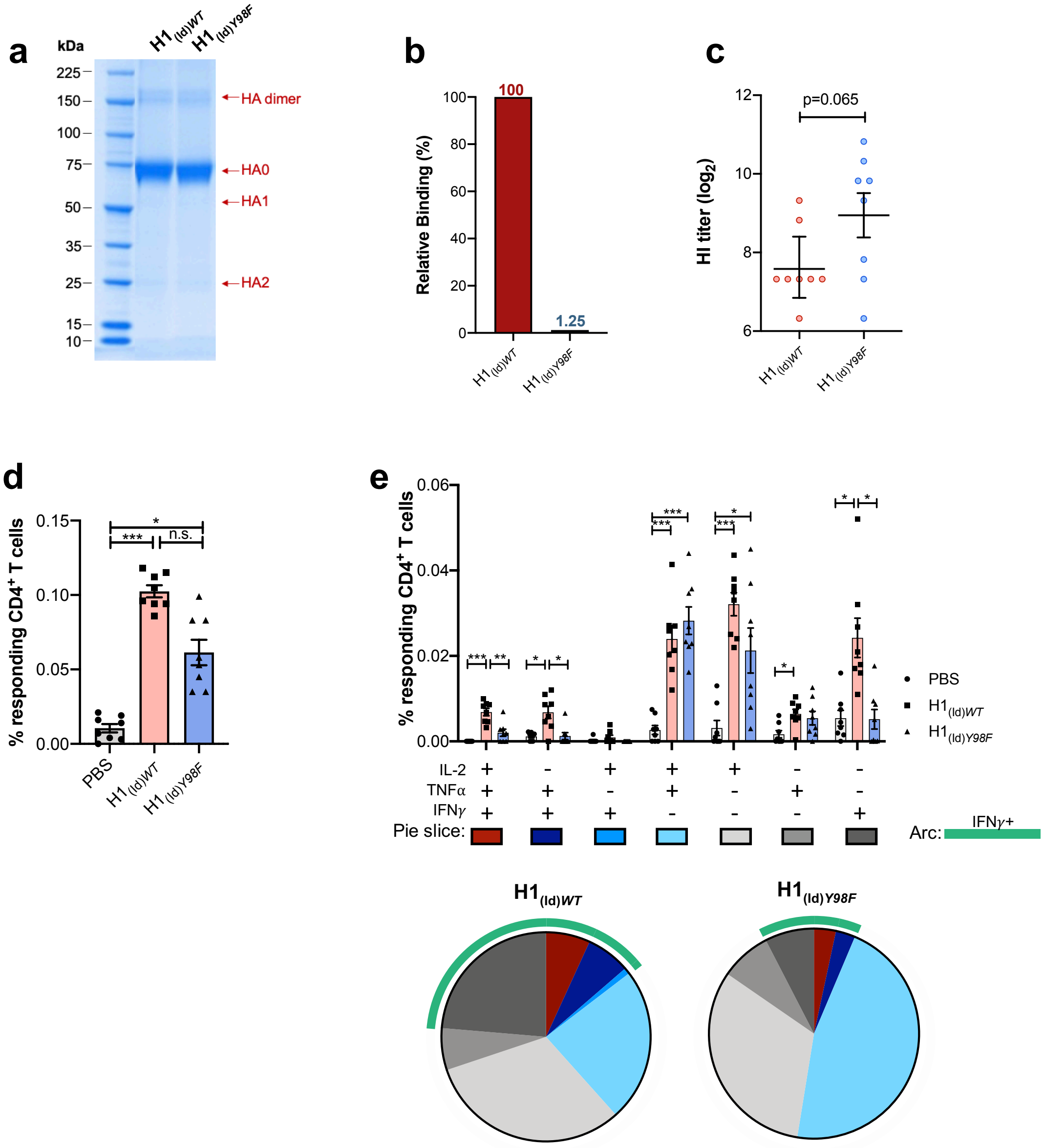

Supplementary figure 3. Plant-based H1*Y98F*-VLP targeting H1 A/Idaho/07/2018 elicits similar patterns of immunity to H1*Y98F*-VLP targeting H1 A/California/07/2009. Wild-type (WT) and Y98F H1-VLPs targeting A/Idaho/07/2018 ($H1_{(Id)WT}$- and $H1_{(Id)Y98F}$-VLPs, respectively) were expressed and purified using the same methods as the H1 A/California/07/2009 VLPs. (a) VLP composition and purity were evaluated by SDS-PAGE of purified leaf digests followed by Coomassie G-250 staining. (b) SA binding was quantified by SPR and data represent relative binding of VLPs to α-2,6 SA captured on a streptavidin-coated chip surface (adjusted for HA content). Female Balb/c mice were vaccinated (IM) with 2 doses (1µg each) of $H1_{(Id)WT}$-VLP or

$H1_{(Id)\mathit{Y98F}}$-VLP 21d apart and were euthanized 28d post-boost. (c) HI titers were increased in mice vaccinated with $H1_{(Id)\mathit{Y98F}}$-VLP but narrowly failed to achieve statistical significance determined by Mann-Whitney test. Splenocytes were restimulated with 2.5µg/mL $H1_{(Id)\mathit{WT}}$-VLP to measure $H1_{(Id)}$-specific $CD4^+$ T cells. (d) Both VLPs elicited a significant population of $CD44^+$ $CD4^+$ T cells expressing at least one of IL-2, TNFα or IFNγ. (e) Individual cytokine signatures for each mouse obtained by Boolean analysis. Background values obtained from non-stimulated samples were subtracted from values obtained following stimulation with $H1_{(Id)\mathit{WT}}$-VLP. Statistical significance was determined by Kruskal-Wallis test with Dunn's multiple comparisons (total response) or two-way ANOVA with Tukey's multiple comparisons (cytokine signatures) (*p<0.033, **p<0.01, ***p<0.001).

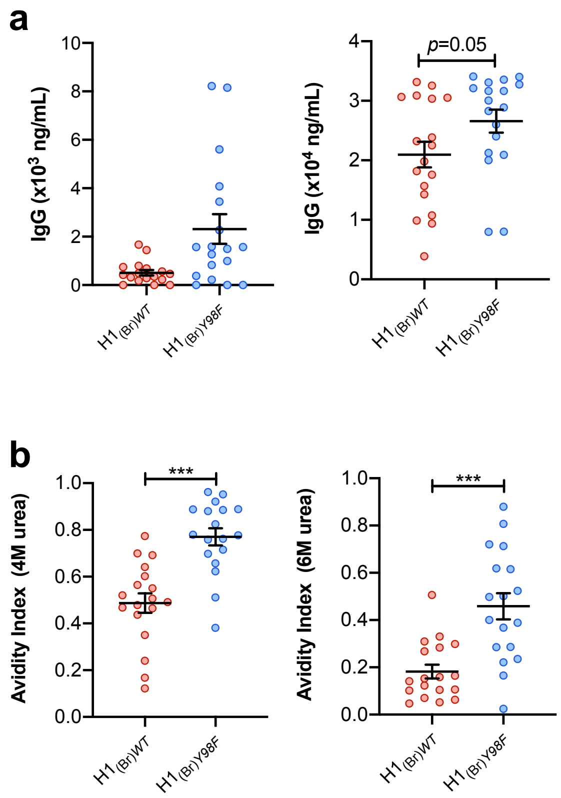

Supplementary figure 4. Cell-culture derived recombinant H1$_{Y98F}$ trimers targeting H1 A/Brisbane/02/2018 elicit similar patterns of humoral immunity to H1$_{Y98F}$-VLP targeting H1 A/California/07/2009. Recombinant wild-type (WT) and Y98F H1 A/Brisbane/02/2018 trimers (H1$_{(Br)WT}$ and H1$_{(Br)Y98F}$, respectively) were purchased from eEnzyme (cat. No. IA-H1-B18WP and IA-H1-B18WPm, respectively). H1$_{(Br)}$ is considered to be antigenically similar to H1$_{(Id)}$ by the WHO and only differs by 3 amino acids in the stem region (WHO vaccine recommendations 2019). Thus, the Y98F mutation was assumed to ablate binding similar to H1$_{(Id)Y98F}$. Female Balb/c mice were vaccinated (IM) with 2 doses (0.5µg each) of recombinant H1$_{(Br)WT}$ or H1$_{(Br)Y98F}$ 21d apart. (a) H1$_{(Br)}$-specific IgG was measured by ELISA on d21 (pre-boost, left panel) and 21d post-boost (right panel). (b) At 21d post-boost, IgG avidity was evaluated by ELISA following incubation with 4M urea (left) or 6M urea (right). Statistical significance was determined by Mann-Whitney test (***p<0.001).

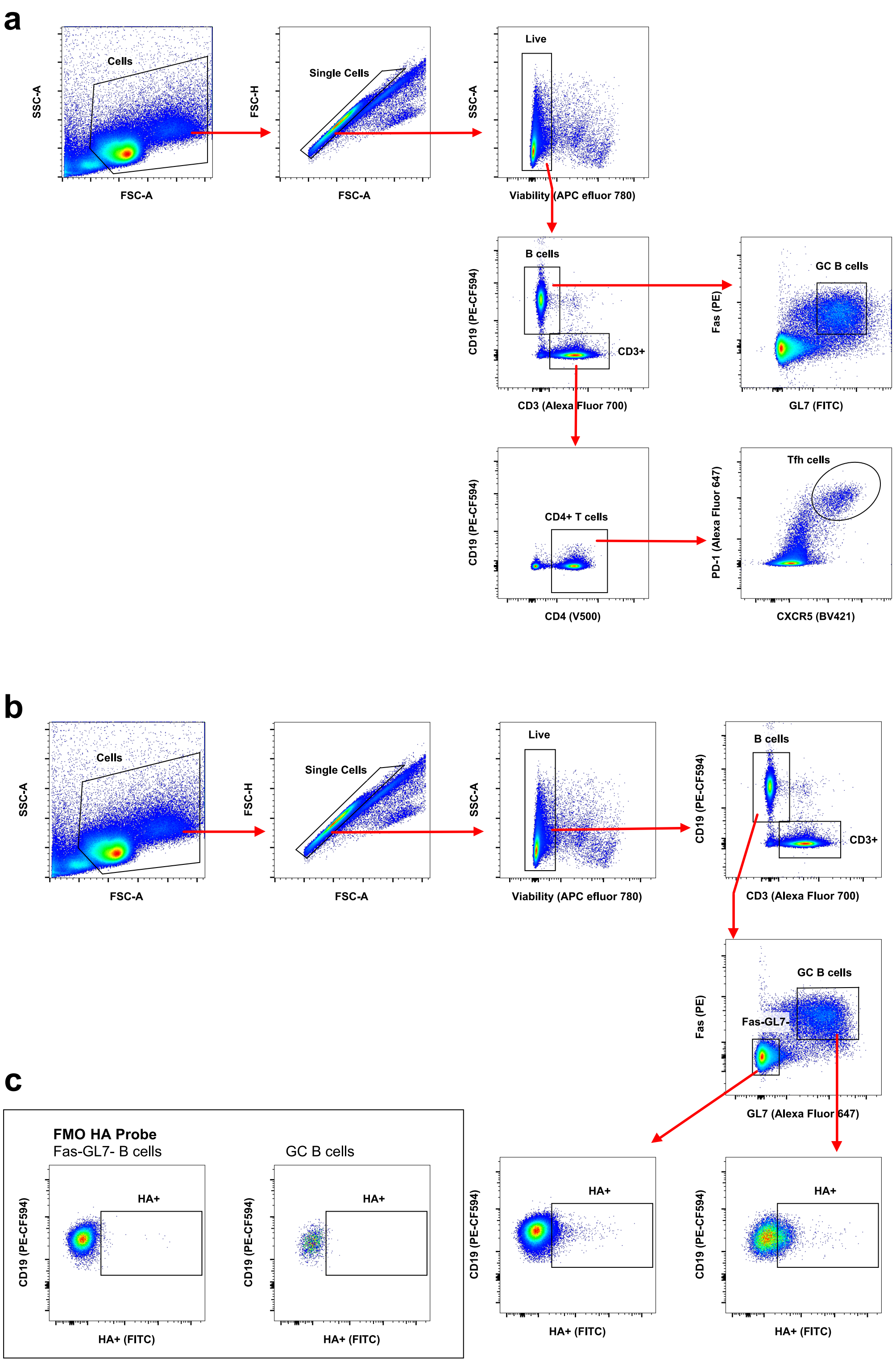

Supplementary figure 5. (legend on the following page)

Supplemental figure 5. Flow cytometry gating strategy to identify germinal center B cells and T_{FH} cells. In all analyses cells were first gated to exclude debris, cell clusters and dead cells. (a) Gating strategy to identify total GC B cells and T_{FH} cells in the draining popliteal LN following footpad injection (presented in figures 3a and 3b, respectively). GC B cells are $CD19^{+}Fas^{+}GL7^{+}$ and T_{FH} cells are $CD3^{+}CD4^{+}CXCR5^{+}PD\text{-}1^{+}$. (b) Gating strategy to identify HA-specific B cells among GC B cells (presented in figure 3c-d) and non-GC B cells ($Fas^{-}GL7^{-}$, presented in supplementary figure 7). Prior to staining, cells were incubated with 1µg/mL $H1_{Y98F}$-VLP as a probe to identify antigen-specific cells. B cells that bound to the probe were identified via staining with anti-H1 FITC. (c) HA+ cells were negligible when the $H1_{Y98F}$-VLP probe was not added prior to staining.

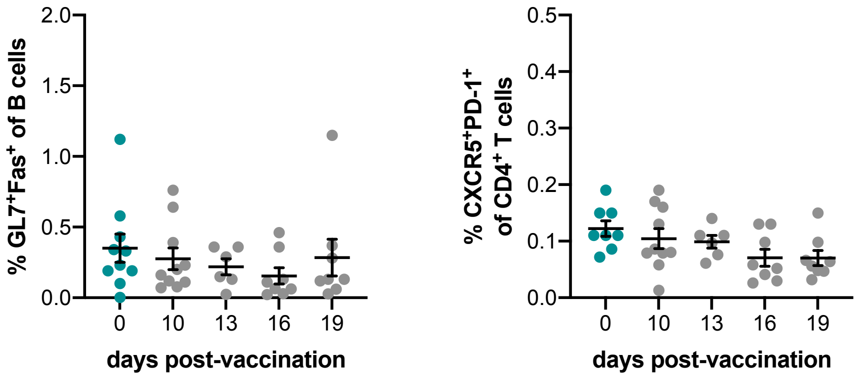

Supplementary figure 6. Footpad injection of PBS does not elicit a GC response in the draining popliteal LN. Mice were injected in the right hind limb footpad with 50µL PBS and draining pLN were collected at indicated time points following injection. Frequencies of (a) $CD19^{+}GL7^{+}Fas^{+}$ GC B cells and (b) $CD3^{+}CD4^{+}CXCR5^{+}PD\text{-}1^{+}$ T_{FH} cells were determined by flow cytometry (mean±SEM). There was no increase in either population compared to pLN collected from non-vaccinated mice (D0, blue).

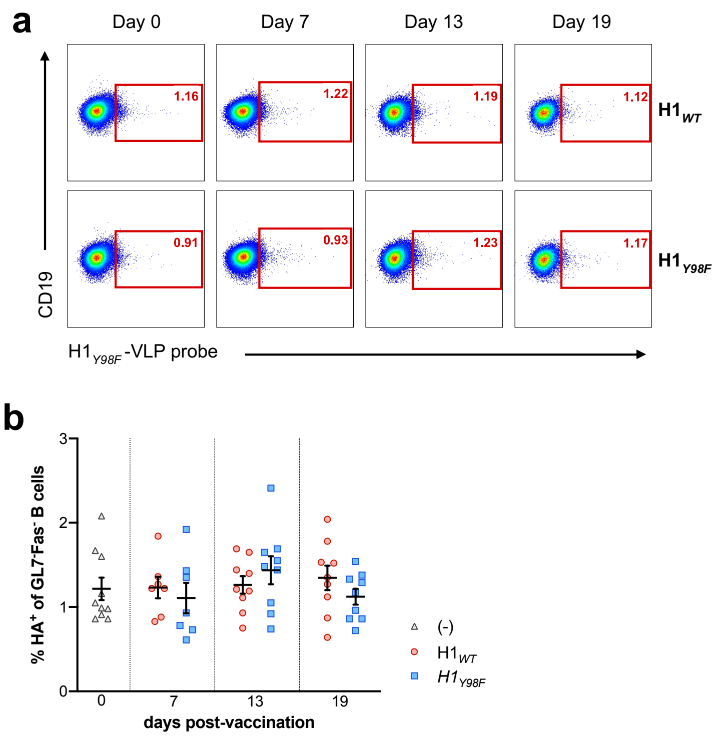

Supplementary figure 7. HA-specific B cells outside of the GC remain near baseline levels following vaccination. Mice were immunized with 0.5µg $H1_{WT}$- or $H1_{Y98F}$-VLP in the right hind-limb footpad and popliteal lymph nodes (pLN) were harvested at indicated time points. (a) Representative plots and (b) mean frequency (±SEM) of HA+ cells among $Fas^{-}GL7^{-}$ B cells.

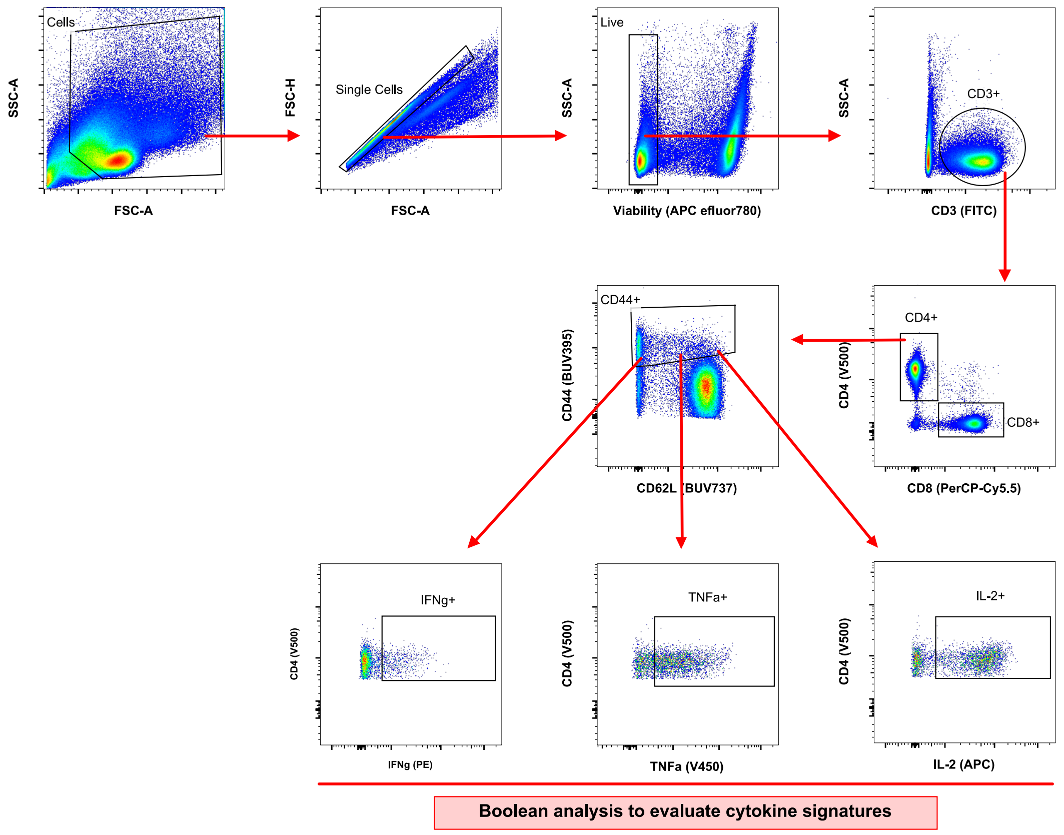

Supplementary figure 8. Flow cytometry gating strategy to identify antigen-specific CD4$^+$ T cells presented in figure 4a-c. Cells were first gated to exclude debris, cell clusters, and dead cells. Antigen experienced CD4$^+$ T cells were distinguished based on expression of CD44. H1-specific CD4$^+$ T cells were identified based on expression of IFNγ, TNFα, and IL-2 following stimulation with H1$_{WT}$-VLP or H1 peptide pool. Boolean analysis was used to evaluate cytokine signatures.

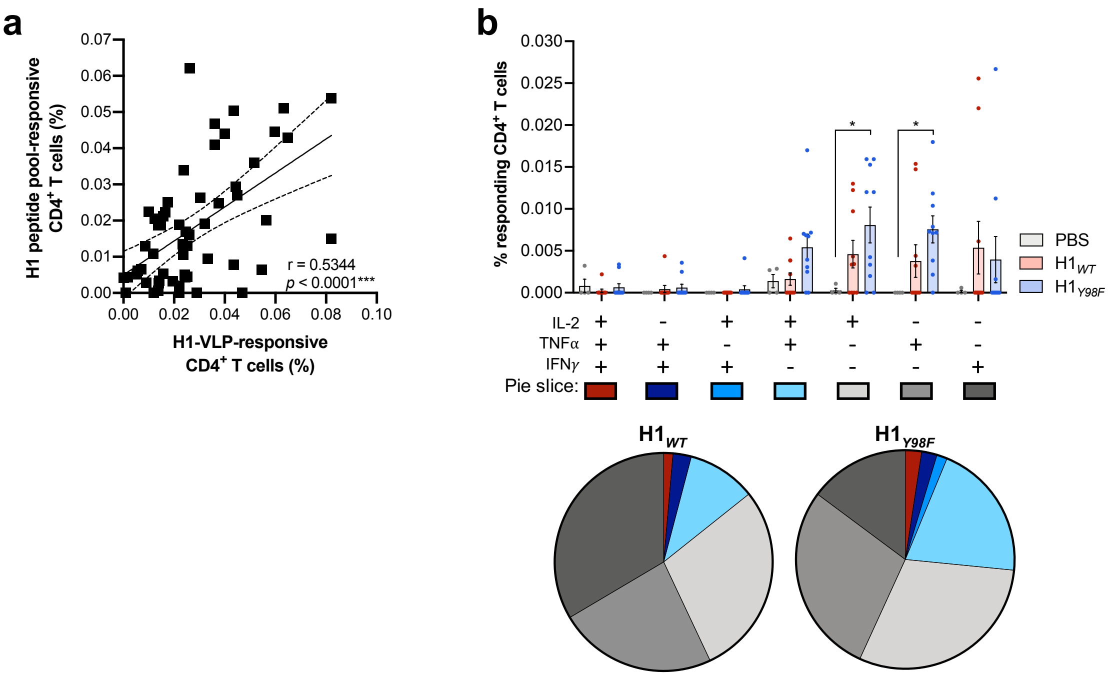

Supplementary figure 9. Stimulation of splenocytes with H1*WT*-VLP and H1 peptide pool results in similar patterns of cytokine expression. Mice were vaccinated with 2 doses (21d apart) with 0.5µg H1*WT*- or H1*Y98F*-VLP. Mice were euthanized 28d post-boost and freshly isolated splenocytes were stimulated with 2.5µg/mL H1*WT*-VLP or a pool of 131 overlapping peptides (15aa) spanning the entire H1 A/California/07/2009 sequence. The frequency of antigen-experienced ($CD44^+$) $CD4^+$ T cells expressing at least one of IL-2, TNFα or IFNγ was determined by flow cytometry. (a) Spearman's rank correlation comparing the frequency of responding antigen-experienced $CD4^+$ T cells following stimulation with H1*WT*-VLP or H1 peptide pool. (b) Individual cytokine signatures for each mouse determined by Boolean analysis of responding $CD4^+$ T cells. The frequency of responding cells in non-stimulated samples were subtracted from frequency of responding cells following stimulation with H1 peptide pool (2.5µg/mL). Statistical significance was determined by two-way ANOVA with Tukey's multiple comparisons (*$p<0.033$, **$p<0.01$, ***$p<0.001$)

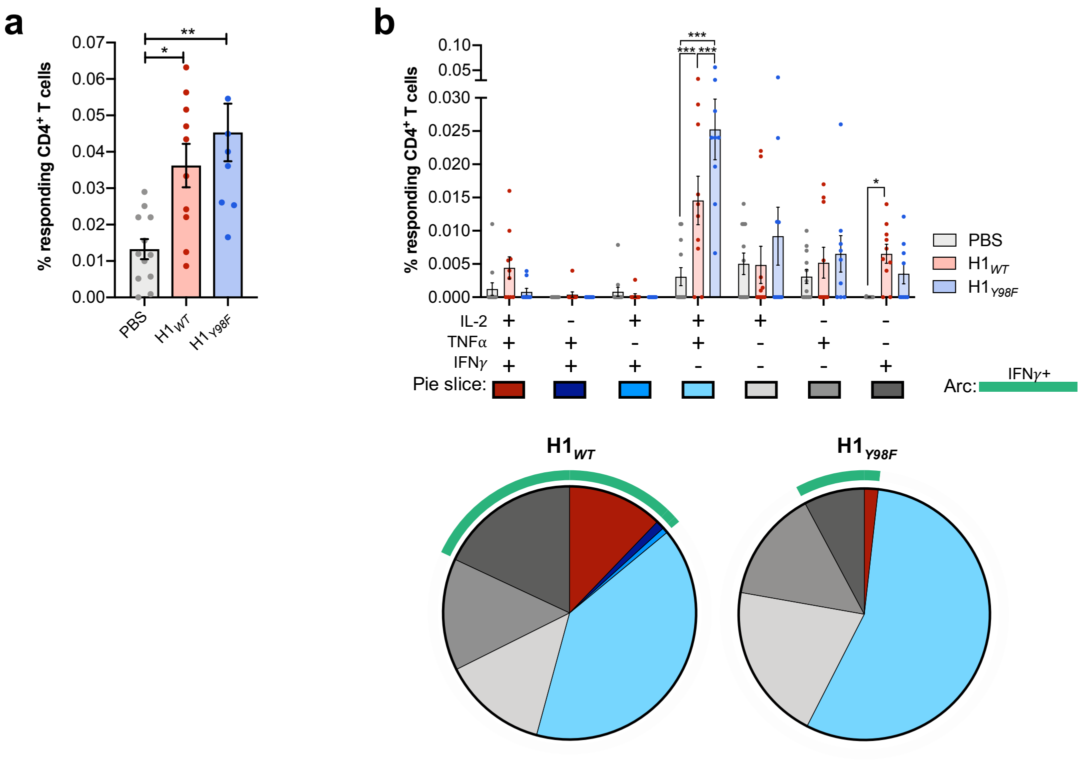

Supplementary figure 10. Increased antigen dose increases the magnitude of the CD4+ T cell response but does not affect cytokine signatures. Mice were vaccinated twice with 3µg H1$_{WT}$- or H1$_{Y98F}$-VLP 21d apart. Spleens were harvested 28d post-boost and splenocytes were stimulated for 18h with 2.5µg/mL H1$_{WT}$-VLP. Antigen-specific CD4^{+} T cells were quantified by flow cytometry. (a) Frequency of CD4^{+} T cells expressing CD44 and at least one of IL-2, TNFα or IFNγ. (b) Individual cytokine signatures for each mouse obtained by Boolean analysis. Background values obtained from non-stimulated samples were subtracted from values obtained following stimulation with H1$_{WT}$-VLP. Statistical significance was determined by Kruskal-Wallis test with Dunn's multiple comparisons (total response) or two-way ANOVA with Tukey's multiple comparisons (cytokine signatures) (*p<0.033, **p<0.01, ***p<0.001).

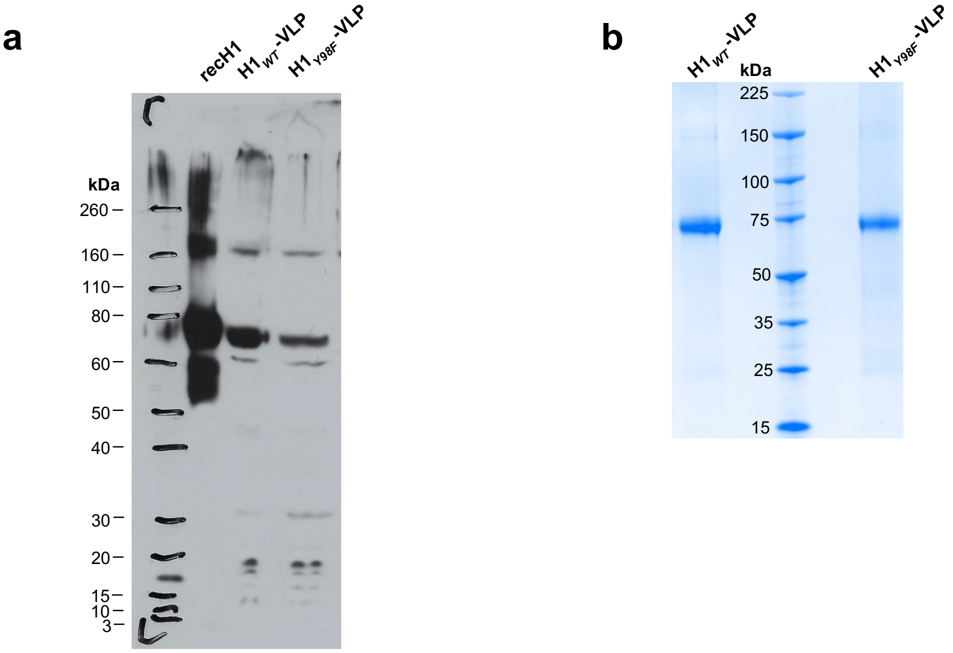

Supplementary figure 11. Unmodified western blot and gel images. (a) Western blot corresponding to figure 1b. (b) Coomassie stained gel corresponding to figure 1c.
