# Supplementary material for: Elimination of receptor binding by influenza hemagglutinin improves vaccine-induced immunity
Source: NPJ Vaccines. 2022 Apr 11;7:42. doi: 10.1038/s41541-022-00463-3 (PMC9001741; doi:10.1038/s41541-022-00463-3)
Supplement: Supplementary file 2 — REPORTING SUMMARY [file 41541_2022_463_MOESM2_ESM.pdf]

nature portfolio

Corresponding author(s): Brian J Ward

Last updated by author(s): Dec 20, 2021

Reporting Summary

Nature Portfolio wishes to improve the reproducibility of the work that we publish. This form provides structure for consistency and transparency in reporting. For further information on Nature Portfolio policies, see our Editorial Policies and the Editorial Policy Checklist.

Statistics

For all statistical analyses, confirm that the following items are present in the figure legend, table legend, main text, or Methods section.

n/a	Confirmed	
☐	☒	The exact sample size (*n*) for each experimental group/condition, given as a discrete number and unit of measurement
☐	☒	A statement on whether measurements were taken from distinct samples or whether the same sample was measured repeatedly
☐	☒	The statistical test(s) used AND whether they are one- or two-sided<br>*Only common tests should be described solely by name; describe more complex techniques in the Methods section.*
☒	☐	A description of all covariates tested
☐	☒	A description of any assumptions or corrections, such as tests of normality and adjustment for multiple comparisons
☐	☒	A full description of the statistical parameters including central tendency (e.g. means) or other basic estimates (e.g. regression coefficient) AND variation (e.g. standard deviation) or associated estimates of uncertainty (e.g. confidence intervals)
☐	☒	For null hypothesis testing, the test statistic (e.g. *F*, *t*, *r*) with confidence intervals, effect sizes, degrees of freedom and *P* value noted<br>*Give P values as exact values whenever suitable.*
☒	☐	For Bayesian analysis, information on the choice of priors and Markov chain Monte Carlo settings
☒	☐	For hierarchical and complex designs, identification of the appropriate level for tests and full reporting of outcomes
☐	☒	Estimates of effect sizes (e.g. Cohen's *d*, Pearson's *r*), indicating how they were calculated

Our web collection on statistics for biologists contains articles on many of the points above.

Software and code

Policy information about availability of computer code

Data collection	Flow cytometry data was collected on a BD LSRFortessa or BD LSRFortessa X20 flow cytometer (BD Biosciences)<br>ELISpot plates were counted and analyzed using an Immunospot S6 plate reader<br>Antibody ELISA plates were read at 450nm on an EL800 microplate reader (Biotek)<br>Cytokine multiplex ELISA plates were read using a Q-View imager (Quansys Biosciences)
Data analysis	Flow cytometry data was analysed using FlowJo software v10 (Treestar, Ashland)<br>Cytokine multiplex ELISA data were analyzed using Q-View software (Quansys Biosciences)<br>Statistical analyses were conducted using Graphpad Prism v9.2.0 software.

For manuscripts utilizing custom algorithms or software that are central to the research but not yet described in published literature, software must be made available to editors and reviewers. We strongly encourage code deposition in a community repository (e.g. GitHub). See the Nature Portfolio guidelines for submitting code & software for further information.

Data

Policy information about availability of data

All manuscripts must include a data availability statement. This statement should provide the following information, where applicable:

- Accession codes, unique identifiers, or web links for publicly available datasets
- A description of any restrictions on data availability
- For clinical datasets or third party data, please ensure that the statement adheres to our policy

All data are available in the main text. Additional information can be requested through the corresponding author.

Field-specific reporting

Please select the one below that is the best fit for your research. If you are not sure, read the appropriate sections before making your selection.

☒ Life sciences ☐ Behavioural & social sciences ☐ Ecological, evolutionary & environmental sciences

For a reference copy of the document with all sections, see nature.com/documents/nr-reporting-summary-flat.pdf

Life sciences study design

All studies must disclose on these points even when the disclosure is negative.

Sample size	Group sizes were selected based experience with similar previous studies. For survival following challenge we used n=8 mice per group and confirmed findings in an independent study using n=4 mice per group. To evaluate viral load and pulmonary inflammation, we conducted two independent experiments with n=4-5 mice/group/time point. For evaluation of long-term antibody response, we evaluated n=7-8 mice per group. For evaluation of GC kinetics, we conducted 3 independent experiments with n=4-6 mice/group/time point (each time point evaluated in 2/3 studies). For evaluation of T cell responses, we conducted two independent experiments with n=5-8 mice/group.
Data exclusions	In the long-term study, one mouse was excluded due to a technical error during vaccination (partial dose). In studies examining GC responses, 1 mouse per group on days 13 and 19 was excluded due to a technical error during vaccination (partial dose).
Replication	All mouse experiments were repeated twice to ensure reproducibility. For ELISA, ELISpot, MN assay and HI assay all samples were run in duplicate. All attempts at replication were successful.
Randomization	All animals were randomly assigned to vaccination groups.
Blinding	Blinding was not performed.

Reporting for specific materials, systems and methods

We require information from authors about some types of materials, experimental systems and methods used in many studies. Here, indicate whether each material, system or method listed is relevant to your study. If you are not sure if a list item applies to your research, read the appropriate section before selecting a response.

Materials & experimental systems

n/a	Involved in the study	
☐	☒	Antibodies
☐	☒	Eukaryotic cell lines
☒	☐	Palaeontology and archaeology
☐	☒	Animals and other organisms
☒	☐	Human research participants
☒	☐	Clinical data
☒	☐	Dual use research of concern

Methods

n/a	Involved in the study	
☒	☐	ChIP-seq
☐	☒	Flow cytometry
☒	☐	MRI-based neuroimaging

Antibodies

Antibodies used

CD16/CD32 (Fc block) - BD 553142
CD3 AF700 (clone 17A2) - Biolegend 100216
CD3 FITC (clone 145-2C11) - eBioscience (Thermo Fisher) 11-0031-86
CD19 PE-CF594 (clone ID3) - BD 562291
Fas PE (clone 15A7) - Thermo Fisher 12-0951-83
GL7 FITC (clone GL7) - Biolegend 144607
GL7 AF647 (clone GL7) - Biolegend 144606
PD-1 AF647 (clone 29F.1A12) - Biolegend 135230
CXCR5 biotin (clone 2G8) - BD 551960
H1(A/California/06/2009) FITC (clone 2F3) - Immune Tech IT-003-001M5-FITC
H1(A/California/06/2009) - Immune tech IT-003-SW
CD4 V500 (clone RM4-5) - BD 560782
CD8 PerCP-Cy5.5 (clone 53-6.7) - BD 551162
CD44 BUV395 (clone IM7) - BD 740215
CD62L BUV737 (clone MEL-14) - BD 612-833
IL-2 APC (clone JES7-5H4) - Biolegend 503810
IFNg PE (clone XMG1.2) - BD 562020
TNFa eFluor450 (clone MP6-XT22) - Invitrogen 48-7321-82

	goat anti-rabbit IgG HRP - Immune technology IT-200-01<br>goat anti-mouse IgG HRP - Southern Biotech 1036-95
Validation	All antibodies were validated by the manufacturer and were titrated prior to use.

Eukaryotic cell lines

Policy information about cell lines

Cell line source(s)	MDCK (NBL-2) - ATCC CCL-34
Authentication	Authenticated by vendor.
Mycoplasma contamination	ATCC cell lines are guaranteed to be mycoplasma negative and no mycoplasma contamination was detected.
Commonly misidentified lines<br>(See ICLAC register)	N/A

Animals and other organisms

Policy information about studies involving animals; ARRIVE guidelines recommended for reporting animal research

Laboratory animals	Female BALB/c mice (8-10 weeks of age) were obtained from Charles River
Wild animals	N/A
Field-collected samples	N/A
Ethics oversight	All animal procedures were carried out in accordance with guidelines of the Canadian Council on Animal Care, as approved by the Animal Care Committee of McGill University.

Note that full information on the approval of the study protocol must also be provided in the manuscript.

Flow Cytometry

Plots

Confirm that:

☒ The axis labels state the marker and fluorochrome used (e.g. CD4-FITC).

☒ The axis scales are clearly visible. Include numbers along axes only for bottom left plot of group (a 'group' is an analysis of identical markers).

☒ All plots are contour plots with outliers or pseudocolor plots.

☒ A numerical value for number of cells or percentage (with statistics) is provided.

Methodology

Sample preparation	Splenocytes and bone marrow immune cells were isolated as previously described (Yam KK, 2015; Yam KK 2017). Popliteal LN were digested with collagenase D (1mg/mL) and DNaseI (10µg/mL) (40 min, 37degC, shaking at 220 RPM) prior to mechanical dissociation as described for spleens.
Instrument	All flow cytometry was conducted using a BD LSR Fortessa or BD LSRFortessa X20.
Software	Data was analyzed using FlowJo software version 10.8.1 (Treestar, Ashland)
Cell population abundance	No sorting was performed.
Gating strategy	Gating strategies are supplied in supplemental figures.

☒ Tick this box to confirm that a figure exemplifying the gating strategy is provided in the Supplementary Information.
